# Unifying Genetic Canalization, Genetic Constraint, and Genotype-by-Environment Interaction: QTL by Genomic Background by Environment Interaction of Flowering Time in *Boechera stricta*

**DOI:** 10.1371/journal.pgen.1004727

**Published:** 2014-10-23

**Authors:** Cheng-Ruei Lee, Jill T. Anderson, Thomas Mitchell-Olds

**Affiliations:** 1 Department of Biology, Duke University, Durham, North Carolina, United States of America; 2 Department of Biological Sciences, Environment and Sustainability Program, University of South Carolina, Columbia, South Carolina, United States of America; 3 Institute for Genome Sciences and Policy, Duke University, Durham, North Carolina, United States of America; Harvard University, United States of America

## Abstract

Natural populations exhibit substantial variation in quantitative traits. A quantitative trait is typically defined by its mean and variance, and to date most genetic mapping studies focus on loci altering trait means but not (co)variances. For single traits, the control of trait variance across genetic backgrounds is referred to as genetic canalization. With multiple traits, the genetic covariance among different traits in the same environment indicates the magnitude of potential genetic constraint, while genotype-by-environment interaction (GxE) concerns the same trait across different environments. While some have suggested that these three attributes of quantitative traits are different views of similar concepts, it is not yet clear, however, whether they have the same underlying genetic mechanism. Here, we detect quantitative trait loci (QTL) influencing the (co)variance of phenological traits in six distinct environments in *Boechera stricta*, a close relative of *Arabidopsis*. We identified *nFT* as the QTL altering the magnitude of phenological trait canalization, genetic constraint, and GxE. Both the magnitude and direction of *nFT*'s canalization effects depend on the environment, and to our knowledge, this reversibility of canalization across environments has not been reported previously. *nFT*'s effects on trait covariance structure (genetic constraint and GxE) likely result from the variable and reversible canalization effects across different traits and environments, which can be explained by the interaction among *nFT*, genomic backgrounds, and environmental stimuli. This view is supported by experiments demonstrating significant *nFT* by genomic background epistatic interactions affecting phenological traits and expression of the candidate gene, *FT*. In contrast to the well-known canalization gene *Hsp90*, the case of *nFT* may exemplify an alternative mechanism: Our results suggest that (at least in traits with major signal integrators such as flowering time) genetic canalization, genetic constraint, and GxE may have related genetic mechanisms resulting from interactions among major QTL, genomic backgrounds, and environments.

## Author Summary

Biological traits often display large amounts of genetic variability as well as genetic correlations among traits. This variability provides the raw material for evolutionary change and may alter the direction of trait evolution under selection. Despite this importance, it is unclear whether the genetic controls of variability in single traits and relationships among multiple traits have related mechanisms. Using the flowering time of a plant species as model, here we performed genetic mapping and identified a locus altering single-trait variability and multi-trait relationships. The effect likely results from the distinct thresholds required by its different alleles to trigger flowering, which can be explained by the interaction among this major locus, the variable genomic backgrounds, and the distinct environments. This view is supported by experiments showing epistatic effects of this major locus on flowering time and expression pattern of the candidate gene. Together, our results show that, at least for traits with major signal integrator genes such as flowering time, the genetic control of single-trait variability and multi-trait relationships may have a common underlying mechanism that may be generalizable to other genes or pathways, mediated by interaction among major loci, genomic backgrounds, and surrounding environments.

## Introduction

Elucidating the genetic basis of quantitative traits enables analysis of the processes that shape trait evolution [1]. Two properties that characterize quantitative traits in a population are mean and (co)variance. Most genetic mapping studies focus on loci that influence trait means, while the genomic regions capable of altering trait (co)variances, in contrast, receive relatively little attention despite the crucial role of trait (co)variances in determining the potential for traits to respond to selection [but see 2]. Although researchers have long recognized the importance of genetic control over phenotypic variance [3]–[6], only recently have analyses begun to map genomic regions or genes responsible for trait (co)variance in organisms such as *Arabidopsis thaliana* [7]–[9], yeast [10], or mouse [11], [12]. In humans, recent studies also have identified variance-controlling loci with biomedical implications [13]–[16].

For a quantitative trait, the phenotypic variation, *V_P_*, can be decomposed as *V_P_* = *V_E_*+*V_G_*+*V_GxE_*+*V_e_*, where *V_E_* is the major environmental variance from different growth chambers or experimental gardens, *V_G_* is the genetic variance, *V_GxE_* stands for the genotype-by-environment interaction, and *V_e_* is the stochastic noise caused by micro-environmental differences or other factors. For experiments across several different major environments, ‘plasticity’ consists of *V_E_* and *V_GxE_*. Within the same major environment (where *V_E_* and *V_GxE_* can be ignored) canalized traits exhibit little phenotypic variation (*V_P_*) [6], and causes of phenotypic canalization can be environmental or genetic. That is, one genotype produces a constant phenotype despite environmental variation (*environmental canalization* reducing *V_e_*), or genetically distinct individuals produce similar phenotypes despite different genetic backgrounds (*genetic canalization* reducing *V_G_*) [6], [17], [18]. Most mapping studies of canalization loci focused on environmental canalization, where the stochastic noise (*V_e_* or similar measures) of inbred lines was often used as a quantitative trait in standard mapping algorithms [7], [9]–[12]; only in a few cases are we aware of attempts to specifically map loci controlling genetic canalization [8], [12]. While Shen *et al.* [8] identified loci whose alleles differ in the genetic variance among inbred lines (*V_G_*), Fraser and Schadt [12] employed separate approaches to map loci controlling genetic canalization and environmental canalization. Here similar to both studies [8], [12], we focus on genetic canalization, which epistatically reduces genetic variance conferred by other genes [4], [6], [17] without changing the extent of molecular polymorphism in the genome. Beyond single traits, in this study we further consider the genetic control of the relationship among multiple traits.

The relationship among traits is often expressed in the form of their genetic variance-covariance matrix, the **G** matrix [19]. The **G** matrix of different traits in the same environment indicates the magnitude of potential genetic constraints, whereas the **G** matrix of the same traits in different environments is algebraically related to the genotype-by-environment interaction component of plasticity (*V_GxE_*, sometimes referred to as GxE below) [20]–[22]. While the term ‘genetic constraint’ may be used more broadly for many different combinations of traits (such as the same trait in different ages) [23], here we use this term only to describe the relationship among different traits in the same environment or age. Genetics may control or alter the magnitude of single-trait genetic variance and the size, shape, or orientation of multi-trait genetic covariance structure (genetic constraint or the genotype-by-environment component of plasticity). While to date several studies are available for the genetic mapping of single-trait genetic canalization, few ecologically or evolutionarily important loci controlling trait covariance structure have been investigated, and it is unclear whether these three attributes of quantitative traits (genetic canalization, genetic constraint, and GxE) have related underlying genetic mechanisms.

Because plants often synchronize their phenology with specific environmental conditions [24], flowering time provides a useful model to investigate these attributes. We examined *Boechera stricta*, an ecological model organism closely related to Arabidopsis [25], [26]. Previously we showed that flowering time is under strong selection in nature [25], and a large-effect phenology QTL (*nFT*) exhibits trade-offs in flowering probability and fitness across different natural environments [27], [28], providing a good model to investigate the interaction between genetics and environments. Here, using phenological traits in this species, we provide a unifying framework by identifying the same QTL that alters the magnitude of genetic canalization, genetic constraint, and GxE, and proposing that the genetic mechanism likely involves major QTL by genomic background by environment interaction effects. We further test the proposed epistasis effect between major QTL and genomic background on plant phenological traits and candidate gene expression.

## Results

### Environment-dependent genetic canalization effect

Using recombinant inbred lines (RIL) between *B. stricta* parents from Colorado and Montana, we performed mapping for loci that affect the among-RIL genetic variance (i.e., genetic canalization) of phenological traits (flowering time and plant size [number of leaves] at flowering) in six different growth chamber environments. We identified three quantitative trait loci (QTL) under the stringent genome-wide significance threshold 0.01 (i.e., the observed Brown-Forsythe value is larger than the genome-wide maximum value from at least 990/1000 permutations) (Table 1, Figure S1). The significance threshold of 0.05 had additional QTL identified, but in this study we only focused on QTL with stronger effects. Two QTL (*BST031941* and *Bst004238*) only had canalization effects on flowering time in one environment (Figure S1, 16 hour days, 25°C, 4 week vernalization; i.e., long days, elevated temperature, short winter). The effects of these two QTL were opposite (Figure S2): the Colorado genotype at *BST031941* on linkage group 2 reduced among-RIL genetic variance of (canalized) flowering time, and the Montana genotype at *Bst004238* on linkage group 7 caused flowering time canalization.

**Table 1:** List of variance-controlling QTL, the largest-effect marker within each QTL, the Brown-Forsythe statistics, *P*-value from genome-wide permutation, and proportion of trait variation (*V_p_*) explained by the difference in mean (*V_m_*) or variance (*V_v_*) between two genotypes.

Trait	Environment	QTL	Marker	Brown-Forsythe	P-value	*V_m_*/*V_p_* (%)	*V_v_*/*V_p_* (%)
Flowering time	12 hour days, 18°C, 4 week vernalization	*nFT*	FT_I	16.30	0.003	20.57	3.04
Flowering time	12 hour days, 18°C, 6 week vernalization	*nFT*	Bst011023	16.59	0.005	18.67	2.10
Flowering time	16 hour days, 25°C, 4 week vernalization	*BST031941*	BST031941	20.60	0	12.84	15.90
Flowering time	16 hour days, 25°C, 4 week vernalization	*nFT*	FT_I	15.86	0	11.35	9.60
Flowering time	16 hour days, 25°C, 4 week vernalization	*Bst004238*	Bst004238	15.14	0.005	6.22	7.32
Flowering time	16 hour days, 25°C, 6 week vernalization	*nFT*	FT_I	24.34	0	18.89	3.20
Flowering leaf number	12 hour days, 18°C, 4 week vernalization	*nFT*	FT_I	22.25	0	22.76	5.92
Flowering leaf number	12 hour days, 18°C, 6 week vernalization	*nFT*	Bst011023	26.82	0	21.33	4.20

**Figure 1:**
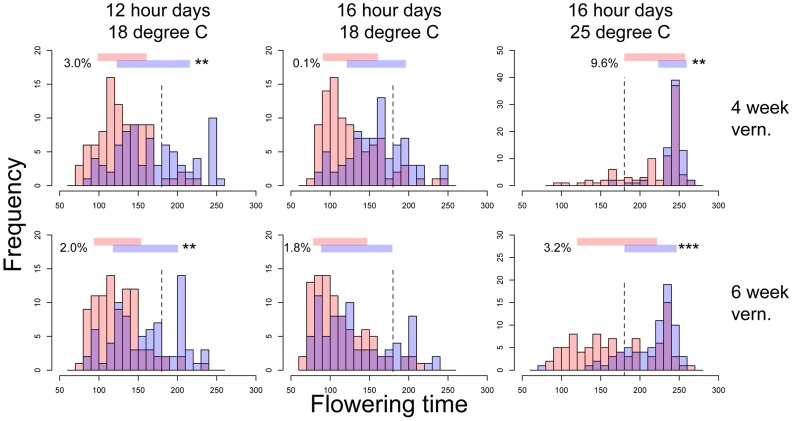
Flowering time distributions of families with the Montana (red bars) or Colorado (blue bars) homozygous genotypes of *nFT* locus in six environments. Panels in each column have the same ambient environment: first column – 12 hour days 18°C, second column – 16 hour days 18°C, third column – 16 hour days 25°C. Panels in each row have the same vernalization treatment: first row – 4 week vernalization, second row – 6 week vernalization. Vertical dashed lines (180 days) separate the two growing seasons in each environment. Above each graph, horizontal bars denote the mean plus or minus one standard deviation for each homozygous genotype, numbers on the left denote percent of total variation explained by the difference in variance of the two genotypes, and asterisks on the right denote genome-wide significance of the difference in variance. * *P*< = 0.05, ** *P*< = 0.01, *** *P*< = 0.001.

**Figure 2:**
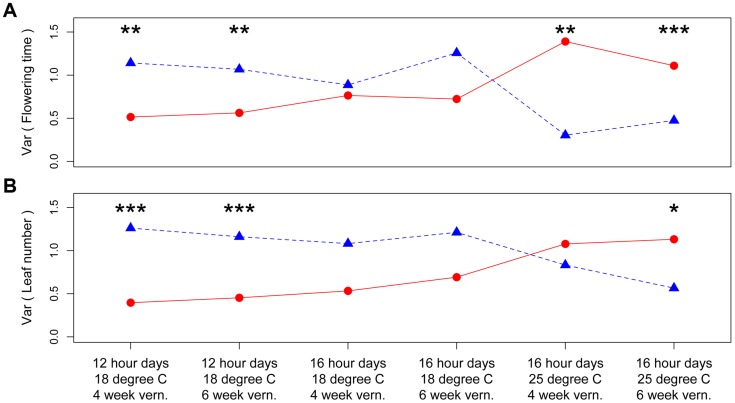
Distinct canalization effects of *nFT* locus across six different environments on standardized traits: (A) flowering time and (B) leaf number at first flowering. Shown are the genetic variances among families homozygous for the Montana (red dots and solid lines) or Colorado (blue triangles and dashed lines) genotypes. In each environment, asterisks above each genotype pair denote the genome-wide significance of difference in variance. * *P*< = 0.05, ** *P*< = 0.01, *** *P*< = 0.001.

The third QTL, *nFT*, had widespread canalization effects in multiple environments. This QTL is syntenic with the region containing flowering time gene *FT* (AT1G65480, hence the name “*near FT*”) in *Arabidopsis thaliana* and is a major QTL influencing *Boechera stricta* phenology, life history and fitness in a broad range of environments [25], [27], [28]. The draft genome assembly from Joint Genome Institute also indicated that the *FT* gene locates within this region. The canalization effect of *nFT* was environment-dependent: under the genome-wide significance threshold of 0.01, its effect was significant for flowering time in four environments (both vernalization lengths in 12 hour 18°C and 16 hour 25°C treatments, Figure 1) and number of leaves at flowering in two environments (both vernalization lengths in 12 hour 18°C, Figure S3). For flowering time, ambient environment significantly influenced the canalization effect, but the duration of vernalization did not (Figure 1). Interestingly, the magnitude and direction of *nFT*'s canalization effect depended on the environment (Figure 2). The Montana genotype reduced the among-RIL genetic variance of (i.e., canalized) phenological traits at 12 hour days and 18°C, but the Colorado genotype had this canalization effect in the 16 hour days, 25°C treatment. Although it is known that the existence and magnitude of genetic canalization vary among environments [29], as far as we know, our study may be the first to show that the direction of canalization effect can be reversed between environments (Figure 2).

**Figure 3:**
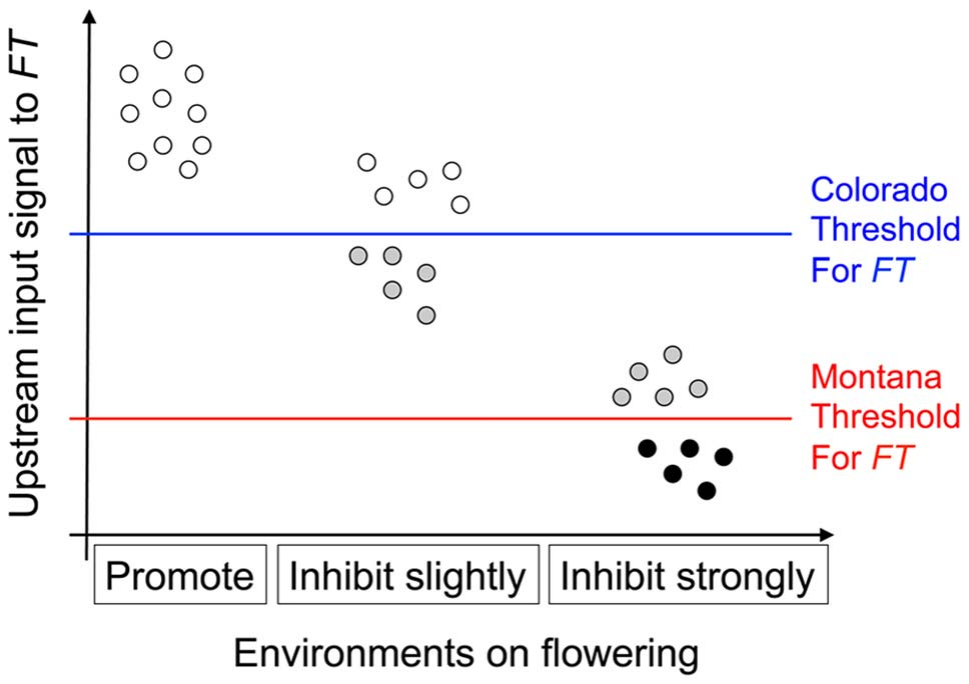
Illustration of the threshold hypothesis, proposing that *nFT*'s reversible canalization effect results from the interaction among *FT* gene, genomic backgrounds, and environments. The horizontal axis denotes three different environments: that promotes flowering (16 hour days 18°C), that slightly inhibits flowering (12 hour days 18°C), and that strongly inhibits flowering (16 hour days 25°C). Here, the same set of genomic backgrounds (the ten dots within each environment, representing different combinations of upstream flowering genes) is replicated among environments, generating different amounts of signal input to *FT* (vertical axis). It is known that the Montana genotype of *nFT* locus accelerates flowering relative to the Colorado genotype in these growth chambers, therefore the Montana genotype has a lower flowering threshold. Based on the specific environment, a genomic background can generate enough signal for both *FT* genotypes to express (white dots), only enough signal for the Montana but not the Colorado genotype (grey dots), or not enough signal for either *FT* genotype (black dots).

Based on these observations, we proposed a ‘*threshold hypothesis*’ to explain the environment-dependent magnitude and direction of *nFT*'s canalization effect (Figure 3), hypothesizing that the *FT* gene may be the causal locus within the *nFT* QTL: In growth chambers, the Montana genotype of *nFT* accelerated flowering in all environments [25]. In addition to *nFT*, these recombinant inbred lines also segregate for other flowering time genes in the genome [25]. Since the *FT* gene within the *nFT* QTL serves as a hub, which promotes flowering after integrating signals from multiple upstream pathways in the *Arabidopsis* flowering time network [30], the environment-dependent canalization effect of *nFT* may be created by the interaction among these factors: 1) different growth chamber environments, 2) distinct genomic backgrounds among RILs created by segregating genotypes of other flowering genes, and 3) the different input-signal threshold needed for either *FT* genotype to trigger flowering (Figures 1 and 3). For plants in the flowering-promoting environment under long days and cool temperature (16 hour days 18°C, mean flowering time at 4 week vernalization = 141 days, and at 6 week vernalization = 122 days), *nFT* did not confer a canalization effect because all genomic backgrounds created high input signals for both *FT* genotypes to initiate flowering. Plants took longer to flower under short days and cool temperatures (12 hour days 18°C, mean flowering time = 147 or 139 days, at 4 or 6 week vernalization, respectively). In these slightly flowering-inhibiting environments, most genomic backgrounds generated lower input signals, which were still enough for the low-threshold Montana genotype to express (predominantly flowering in the first season, within 180 days), whereas a portion of families with the Colorado genotype did not flower until the second growing season because many genomic backgrounds did not generate enough input signals for the high-threshold Colorado genotype (Figures 1 and 3). This resulted in higher variance among *nFT* Colorado homozygotes, such that Montana was the canalization genotype in these environments. Finally, flowering was significantly delayed at elevated temperatures (16 hour days 25°C, mean flowering time at 4 week vernalization = 229 days, at 6 week vernalization = 190 days). In these flowering-inhibiting environments where most genomic backgrounds generated low input signal, some genomic backgrounds still had enough signal for the Montana genotype to flower in the first season, but some families with Montana genotype flowered only in the second season (Figure 1 and Figure 3). The majority of families homozygous for the Colorado genotype, however, did not flower until the second season due to *nFT* Colorado genotype's high threshold. Therefore, the Colorado genotype canalized the onset of reproduction in this environment.

### QTL altering the covariance structure among traits

**Figure 4:**
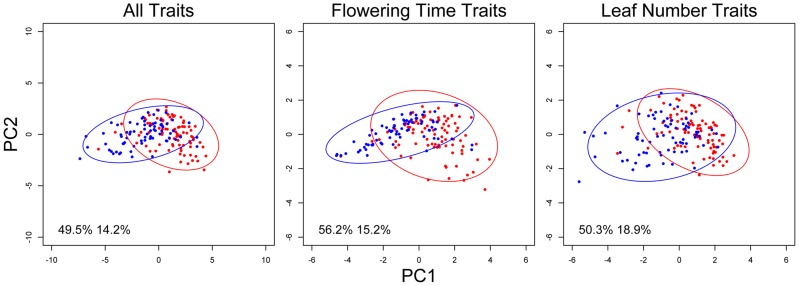
*nFT* alters the covariance structure of multiple phenological traits. Each dot represents the trait values of one recombinant inbred family, and an ellipse represents the 95% confidence region of the covariance matrix defined by an *nFT* homozygous genotype. Montana genotype: red dots and ellipse. Colorado genotype: blue dots and ellipse. Numbers in each graph represent proportional variation explained by PC1 or PC2 of all families, respectively.

While genetic canalization concerns the variance of single traits, the covariance structure of multiple traits may also be affected by genetic elements that canalized some traits but not others. The environment- and trait-dependent canalization effect of *nFT* therefore suggests that it may affect trait covariances (the **G** matrix; the pairwise genetic correlations were reported in Table S1). Indeed, we identified *nFT* as the strongest QTL altering the size (Box's M [31]) and orientation (the angle between the first principal component G_max_) of **G** matrices in different trait-by-environment combinations: flowering time in six environments, leaf number at flowering in six environments, and the combination of all 12 traits (Figure S4). The Krzanowski index method (see Materials and Methods) [32], [33], on the other had, only identified significant *nFT* effect on the covariance structure of flowering time between the six environments but not for plant leaf number at flowering. In contrast to G_max_ angle, the Krzanowski method is likely conservative because it compares the angular difference between subspaces formed by many dimensions, some of which may explain little variation. Figure 4 indicated the difference in size, shape, and orientation between the **G** matrices from the two *nFT* genotypes, and the respective trait loadings on each axis were reported in Table S2. Another QTL, *BST031941*, was also mapped by the Box's M method for the six-flowering-time data set (Figures S4 and S5).

**Figure 5:**
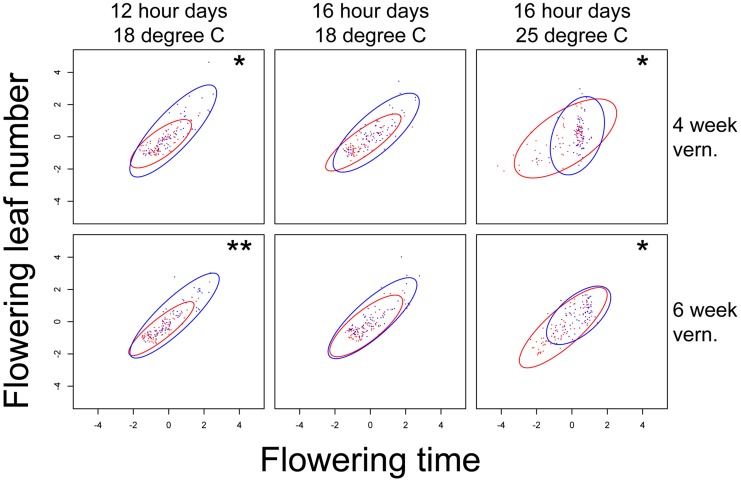
*nFT* effect on the structure of covariance between standardized flowering time and leaf number when flowering in each of the six environments. Asterisks on the upper right of each graph denote genome-wide significance for the Box's M method (ellipse size). *nFT* has no significant effect in the G_max_ angle method (ellipse orientation) in any environment. Montana genotype: red dots and ellipse. Colorado genotype: blue dots and ellipse. * *P*< = 0.05, ** *P*< = 0.01.

We next mapped QTL controlling the **G** matrix between flowering time and leaf number in the same environment (indicating potential genetic constraint) and between the same traits in different environments (indicating the magnitude of the genotype-by-environment interaction component of plasticity). Again, *nFT* and *BST031941* were the only QTL influencing most trait pairs. Similar to the canalization result for univariate traits (above), *nFT* altered the genetic covariance structure between flowering time and leaf number only in both vernalization lengths of two ambient environments: 12 hour days, 18°C and 16 hour days, 25°C, but not 16 hour days, 18°C (Figure 5). The *nFT* effects, however, were not strong (Figure 5) and only significantly influenced the relative size (Box's M) but not the orientation (G_max_ angle) between two **G** matrices in each case. *BST031941*, on the other hand, had effects only in environment 16 hour days, 25°C, 4 week vernalization and altered both the size and orientation of the **G** matrix (Figure S6), consistent with the previous observation that its canalization effect on flowering time only existed in this environment (Figure S1 and S2).

**Figure 6:**
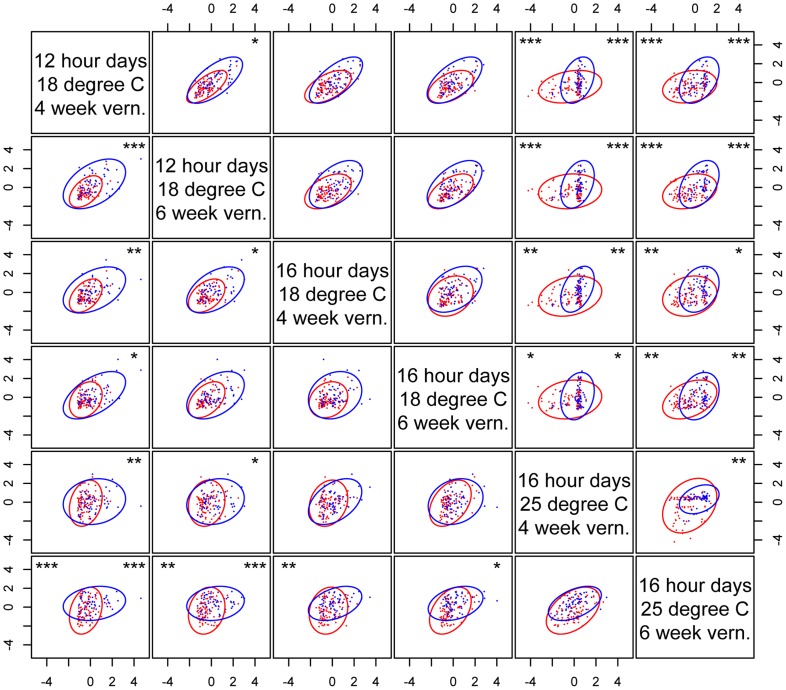
*nFT* effect on the cross-environment plasticity of standardized phenological traits. Each graph shows the relationship of the same trait (above the diagonal – flowering time; below diagonal – leaf number when flowering) between pairs of environments. Montana genotype: red dots and ellipse. Colorado genotype: blue dots and ellipse. In each graph, asterisks in the upper right denote genome-wide significance for the Box's M method (ellipse size), and asterisks on the upper left denote genome-wide significance for the G_max_ angle method (ellipse orientation). * *P*< = 0.05, ** *P*< = 0.01, *** *P*< = 0.001.

Finally, *nFT* influenced the magnitude of GxE (genotype by environment) interactions (Figure 6). For many trait pairs, *nFT* had significant effects on both the size and orientation of the covariance matrices, especially in the comparison between chambers 12 hour days 18°C and 16 hour days 25°C, where *nFT* had significant canalization effect with different directions on univariate traits (Figure 1 and S3). The *BST031941* QTL had a significant effect on GxE for flowering time only in the comparison between 16 hour days, 25°C, 4 week vernalization and all other environments (Figure S7). In addition, the **G** matrices in each comparison differed in both size and orientation. This again is consistent with *BST031941*'s environment-dependent effect described earlier.

### *nFT* epistatic effects in heterogeneous inbred families (HIF)

Since *nFT* influenced the genetic variance and covariance of phenological traits, we hypothesized that this QTL might interact epistatically with other flowering time genes in the genome and alter the magnitude of their effects, as predicted by our ‘threshold hypothesis’. Our previous study did not identify significant epistatic QTL in these growth chambers [25], nor did our re-analyses identify significant interaction effect between *nFT* and other QTL (Table S3). These results, however, did not contradict our prediction, since the threshold hypothesis emphasized the interaction between major QTL (*nFT*) and the cumulative effect from upstream genes (the genomic background), and epistasis between *nFT* and individual upstream QTL may be too weak to detect in the previous experiment.

**Figure 7:**
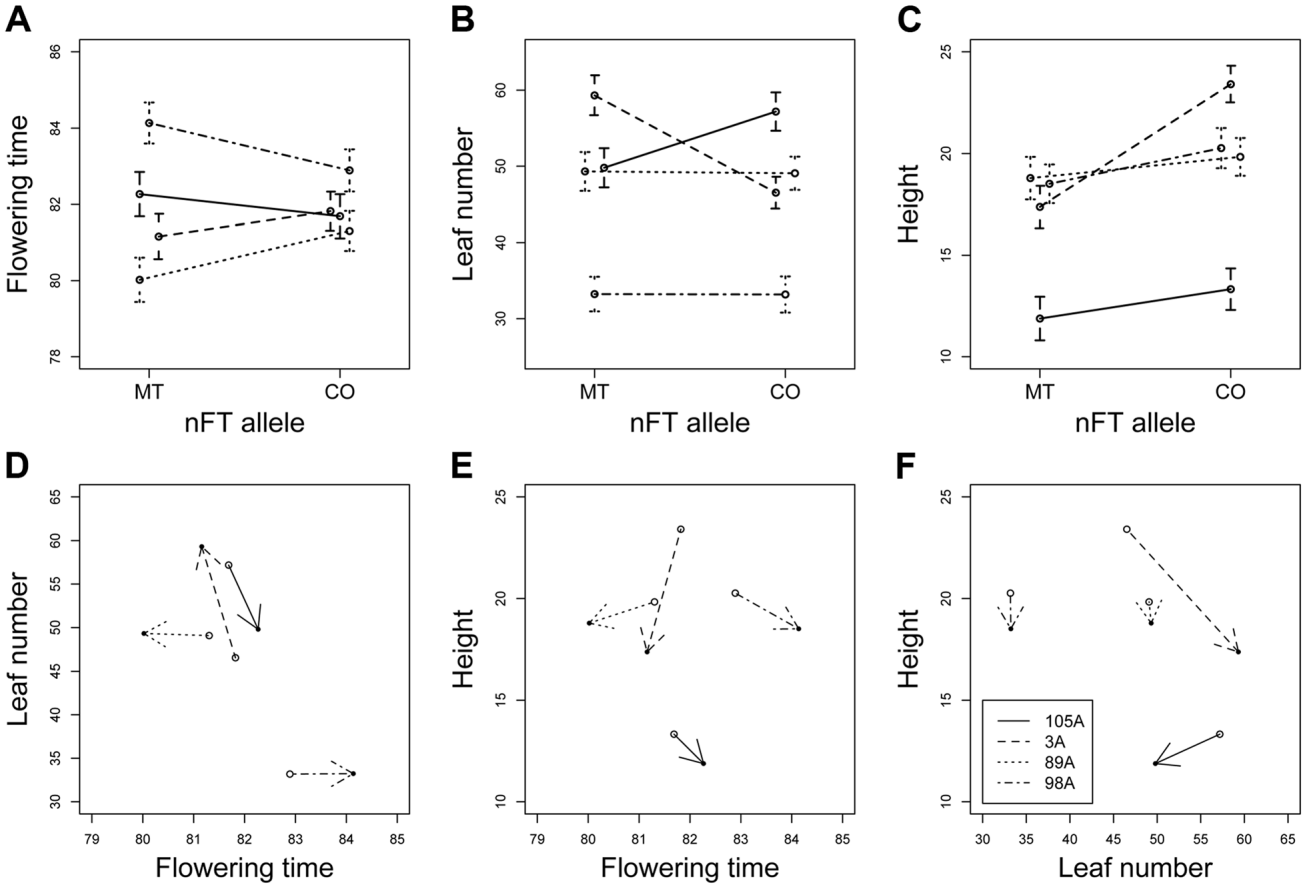
Reaction norms showing the *nFT* by genomic background epistatic effects for three univariate traits (A to C) and three multivariate trait pairs (D to F) in the heterogeneous inbred family (HIF) greenhouse experiment. Lines represent the four HIFs and connect the Montana and Colorado *nFT* homozygous genotypes within each family. Panels A to C show the mean ±1 SE, and epistasis is indicated by the difference in line slopes. In panel D to F, within each family, the arrow points from Colorado to Montana genotype, and epistasis is signified by both the difference in line slopes and arrow directions. Standard error of each *nFT* genotype within each family is shown in panel A to C and is excluded in panel D to F for graph clarity.

To test the epistatic effect between *nFT* and genomic background, we conducted greenhouse experiments using several heterogeneous inbred families (HIF). Each family had almost identical genomic background and segregated for the two *nFT* genotypes, and the use of several such families allowed the test for genomic background by QTL epistatic effects (Figure S8). Univariate analyses revealed *nFT* by genomic background (HIF) interactions for all three traits in the greenhouse environment (flowering time, leaf number and height at flowering, Table 2 and Figure 7 A to C). These effects remained significant after sequential Bonferroni correction. Previously, we detected significant *nFT* effects on mean flowering time in F6 recombinant inbred lines [25]. Here, analyses in the HIF experiments showed significant epistasis but not main *nFT* effects on flowering time. This difference likely reflects the complex effects of *nFT* that depend on genetic background (see Discussion) and is consistent with the idea that the additive effect of a QTL depends on other epistatic genes in the genome [34]. There was, however, a main effect of *nFT* QTL on height at flowering: Montana homozygotes at *nFT* are shorter at flowering than Colorado homozygotes.

**Table 2:** Mixed-model ANOVA for the heterogeneous inbred family (HIF) experiment on phenological traits and *FT* gene expression in the greenhouse environment.

Factor^a^	Flowering time		Flowering leaf number		Flowering height		*FT* gene expression	
	Statistic^b^	*P* value	Statistic	*P* value	Statistic	*P* value	Statistic	*P* value
*nFT*	F_1,686_ = 0.01	0.925	F_1,686_ = 0.70	0.403	F_1,686_ = 14.31	<0.001	F_1,16_ = 18.37	<0.001
HIF	F_3,81_ = 15.63	<0.001	F_3,81_ = 32.49	<0.001	F_3,81_ = 25.75	<0.001	F_1,16_ = 9.33	0.008
*nFT* * HIF	F_3,686_ = 3.54	0.015	F_3,686_ = 5.94	<0.001	F_3,686_ = 3.05	0.028	F_1,16_ = 17.20	<0.001
Family [nested within *nFT* and HIF] (random)	?^2^_1_ = 10.3	<0.001	?^2^_1_ = 25.8	<0.001	?^2^_1_ = 124.3	<0.001	?^2^_1_ = 0.6	0.439
Block (random)	?^2^_1_ = 50.1	<0.001	?^2^_1_ = 0	1	?^2^_1_ = 10.9	<0.001	-	-

a. *P* values of random effects are calculated from likelihood ratio tests between models with and without these effects.

b. Subscript numbers after *F* denotes numerator and denominator degrees of freedom, and subscript numbers after χ^2^ denotes degree of freedom of likelihood ratio test.

In multivariate analysis (MANOVA) simultaneously treating all three traits as response variables, there were significant main effects for *nFT* (*P* = 0.024) and genomic background (*P*<0.001), as well as *nFT* by genomic background interaction effect (*P* = 0.005). Figure 7 D to F show *nFT* by genomic background reaction norms for each pair of traits, demonstrating this epistatic effect.

**Figure 8:**
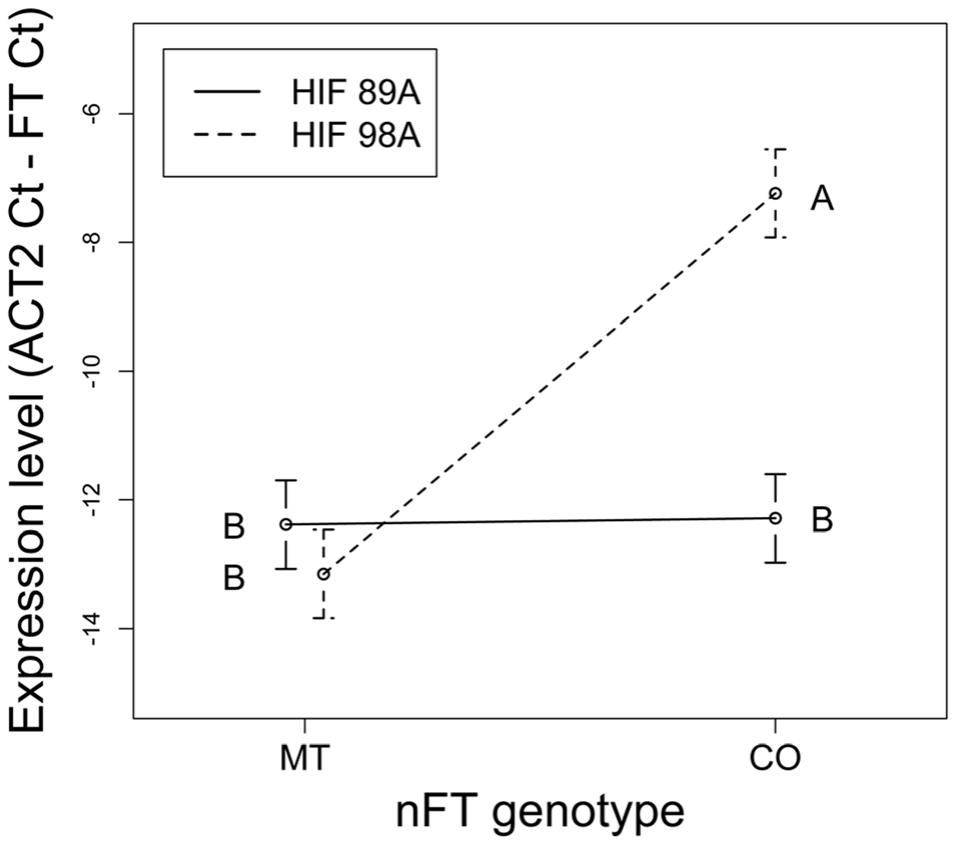
Reaction norms showing the *nFT* by genomic background epistatic effects for expression level of the *FT* transcript. Lines represent the two HIFs and connect the Montana and Colorado *nFT* genotypes within each family (mean ±1 SE). Epistasis is signified by the difference in line slopes. Capital letters show the contrast between all four *nFT* genotype – HIF combinations, and *FT* gene has significantly higher expression in the *nFT* Colorado genotype with HIF 98A genomic background.

While our ‘threshold hypothesis’ focused on the effect of the *FT* gene, the HIF phenotypic experiments only showed effects of the *nFT* QTL. To further test this prediction that the *FT* gene interacts epistatically with other flowering genes in the genome, we used the same HIF experimental design (Figure S8) to test the *nFT* by HIF (genomic background) interaction effect on expression of the *FT* transcript, using two HIF backgrounds. This interaction effect was highly significant (Table 2). While the Montana genotype had low expression in both genomic backgrounds, the Colorado genotype had significantly higher expression in HIF 98A than in HIF 89A (Figure 8). In HIF 89A, both *nFT* genotypes conferred low *FT* gene expression, while in HIF 98A the Colorado *nFT* genotype conferred higher *FT* gene expression than the Montana genotype (Figure 8). This observation is consistent with the threshold hypothesis that the genetic variation of other flowering genes in the HIF 89A background does not generate enough input signals for either *FT* genotype to express. On the other hand, in the greenhouse the HIF 98A genomic background generated enough input signals for the Colorado *FT* genotype to express but not enough for the Montana genotype. In addition, we found a highly significant association between flowering phenotype (presence of visible flowering buds) and quantitative expression of the *FT* transcript in individual plants (*F*_1,17_ = 9.72, *P* value = 0.006). This supports that complex trait variation at the *nFT* QTL may be functionally mediated by the *FT* locus itself.

## Discussion

The genetic basis and evolutionary history of canalization in one trait, genetic constraint among multiple traits, and genotype-by-environment interaction (GxE) across different environments are active foci for research in evolutionary genetics [2]. While many genetic mapping studies are available for single-trait canalization, the majority concerned environmental canalization [7], [9]–[12], and only in a few studies are we aware of mapping for genetic canalization [8], [12]. Here we used similar approaches to previous studies for single-trait genetic canalization [8], [12], but extended our analysis beyond single traits and focused on the QTL altering trait covariance structures. We provide a unifying framework for these mechanisms in the flowering time of *B. stricta* by showing how one QTL (*nFT*, which contains the floral integrator gene *FT*) influences all three attributes for phenological traits of *Boechera stricta*. *nFT* influences single-trait genetic canalization, and the magnitude and direction of its effects can be reversed across environments. *nFT*'s environmentally dependent and reversible effect in one trait, when extending to multiple traits, creates the distinct patterns of covariance structure among different traits in the same environment (altering the magnitude of genetic constraint) or among the same traits in different environments (altering the magnitude of GxE). We propose a ‘threshold hypothesis’ (see Results and Figure 3) to explain this pattern, and the hypothesis is supported by the significant *nFT* by genomic background epistatic effects on phenological traits and transcriptional variation of the candidate *FT* locus in the heterogeneous inbred family (HIF) experiment, echoing studies showing the prevalence of epistasis in both trait and molecular evolution [34]–[41].

### The threshold hypothesis

In this study we proposed a ‘threshold hypothesis’ to explain the environment-dependent genetic canalization effects of *nFT* on flowering time, and the concept is illustrated in Figure 3. This hypothesis focuses on three components of flowering time regulation: the downstream floral signal integrator gene *FT*, the genomic backgrounds with different combinations of polymorphic upstream genes (each generating small input signals to *FT*), and multiple growth chamber environments that also generate different levels of input signals. In this hypothesis, the activation of *FT* expression (which then triggers flowering) depends on whether the upstream input signals, which vary with different combinations of genomic backgrounds and environmental stimuli, exceed the genotype-specific threshold of *FT*. The interaction among major threshold gene, genomic backgrounds, and environmental stimuli therefore triggers the environment-dependent canalization effect of flowering. The threshold model may also help explain how discrete and large-effect phenotypic changes may be caused by continuous and small-effect upstream genetic mechanisms.

In this simple threshold hypothesis, the effect of the genomic background on the initiation of *FT* expression is binary: input signals generated by different backgrounds or environments are either below or above the threshold for *FT* alleles to express. In a previous study of *Arabidopsis thaliana*, Welch *et al.* [42] used sigmoid functions to model genes in the flowering time pathway. These sigmoid functions model the relationship between quantitative upstream input signals and the response of downstream genes, and these models can be viewed as the quantitative generalization of our threshold hypothesis (see details in Figure S9). While in Welch *et al.* [42] the wild-type allele of each gene was assigned a sigmoid function and knockouts always have zero output, in our case the two *FT* alleles with different thresholds simply have different response curves (Figure S9). The threshold hypothesis is therefore supported by previous studies and may be applied to genes or networks that can be modeled with continuous functions.

Our threshold hypothesis also echoes the recent idea that cryptic genetic variation (CVG) is not ‘mechanistically special and mysterious’ [43], but instead that genetic effects are conditional on other genes (epistasis or dominance) or on the environment (GxE), and CVG can be viewed as ‘conditionally neutral genetic variation’ [43]. It is worth emphasizing that the mechanism underlying genetic canalization effects of *nFT* and the well-known *heat shock protein 90* (*Hsp90*) [29], [44] may be different. Hsp90 has a specific function as a protein chaperone [45]–[47], whose expression pattern may be independent of the proteins it helps folding. On the other hand, expression of the *FT* gene is trigged by signals from upstream genes. As a consequence, we predict that distinct input-signal requirements for *FT*'s different genotypes may generate QTL by genomic background interaction effects on phenological traits and on its own expression pattern, which is supported by our HIF experiments. Therefore, in contrast to *Hsp90*, whose specific function may not easily be extrapolated to most genes or pathways, our threshold hypothesis and the case of the *FT* gene in the flowering time pathway may represent an alternative mechanism for other cases of canalization, genetic constraint, and genotype-by-environment interaction in traits with major signal integrators such as flowering time.

In fact, a similar idea has been proposed and tested previously [48]–[50]. Siegal and Bergman [48] modeled the effect of biological networks on trait canalization and showed that canalization is ‘an inevitable consequence of complex developmental-genetic processes’ that does not require the force of stabilizing selection or a specific molecular function such as *Hsp90*. Further studies supported this model by showing that gene knock-outs can affect both genetic and environmental canalization of yeast gene expression [49] as well as phenotypes [50]. More importantly, they showed that ‘capacitors’ (canalization genes) are more likely to be network hubs [50], like the *FT* gene in this study. While these previous studies focus on artificial gene knockouts, here we provide an example of trait canalization from natural variation for ecologically important traits.

Different from other modeling studies, in this study our example of the threshold hypothesis separates the growth chambers into three categories (flowering promoting, slightly inhibitory, and strongly inhibitory) instead of by specific environmental factors (day length, ambient temperature, or vernalization length). For flowering time, pathways responding to different environmental signals converge at floral signal integrator genes such as *FT*, the current focus of the threshold hypothesis, and therefore it is more straightforward to classify environments based on their effect on flowering promotion (the horizontal axis in Figure 3). This is analogous to studies that classify research sites by their effect on plant growth or crop yield [51] and allows modeling flowering time variation without addition information on the expression of upstream genes. We recognize that the threshold hypothesis represents a simplification of the underlying continuous biological process into a qualitative factor (whether plants flower in the first season or not), and future work may explicitly consider the effect of individual environmental factors to allow detailed quantitative modeling of flowering time.

### *nFT* controls univariate trait genetic variance

The link between epistasis and genetic canalization of flowering time has been established in the model plant *Arabidopsis thaliana*. Stinchcombe *et al.* [52] observed that the latitudinal cline in flowering time only exists in genotypes with the wild-type functional allele of *FRIGIDA* but not for the deletion allele (*FRI^Δ^*), and the flowering time of accessions bearing the deletion allele is canalized. This canalization effect is caused by epistasis between *FRIGIDA* and *FLC* [37], where the canalizing *FRI^Δ^* allele suppressed the effect between different alleles of *FLC*. Similarly, in our study we identified *nFT* as the canalization locus and demonstrated the epistatic effects in the HIF experiment, where the different *nFT* genotypes alter the phenotypic effect of different genomic backgrounds (specific allelic combinations of other flowering genes). In addition, as in other cases of canalization genes [29], we also find that *nFT*'s canalization effect depends on the environment.

The adaptive value of canalization has received considerable attention [5], [6]. Theoretical analyses have modeled the conditions under which canalization will evolve [17], [53], [54] (but see [48]–[50]), and empirical studies in *Drosophila* have shown that traits with higher fitness effects have greater canalization [55], [56]. Rutherford *et al.* [29] suggested that the wild-type canalizing allele of *Hsp90* gene in *Drosophila* may be favored because it buffers against potentially unfit background genetic variation. On the other hand, the non-canalizing allele might be favored when a trait is under directional or disruptive selection. Under both scenarios, however, the allelic polymorphism in canalization genes will be eliminated if trait canalization is universally favored or disfavored by natural selection. Our results provide a mechanism where the polymorphism of canalization genes can be maintained. Because which *nFT* genotype has the canalization effect depends on specific environments, different genotypes may be favored under distinct environments even with consistent natural selection for or against trait canalization, beyond mutation-selection balance [17]. For example, if natural selection favors flowering time canalization, in populations under 12 hour days 18°C the Montana genotype would be favored, and the Colorado genotype would be favored under 16 hour days 25°C because they are the canalization genotypes in these respective environments.

The environmental dependence of *nFT* canalization may also maintain the molecular polymorphism of other flowering genes in the genome. Canalization may change the selective influence on other genes by suppressing the genetic variation expressed in traits [18], [57]. In the case of *heat shock protein 90* (*Hsp90*), the wild type allele buffers the phenotypic effect of potentially deleterious mutations in the genome, thereby reducing the force of purifying selection on these mutations [29], [44]. The accumulation of cryptic molecular variation in the genome may provide additional evolutionary potential [18] once the canalization effect is disrupted. The case of *Boechera stricta* flowering time, however, may be more complex due to the *nFT* by environment interaction effect on canalization. Even if the selection force on flowering time is identical across populations, other flowering loci in the genome may still be subject to different types and magnitude of natural selection depending on local environment and *nFT* genotype. Such variation in selection may in turn influence the molecular polymorphism of other flowering time genes.

In this study, flowering time was defined as the days elapsed since initial vernalization, excluding the duration of the second vernalization for plants that did not flower in the first growing season. Since the time of vernalization simulated ‘winter’ conditions with little plant growth, this approach quantifies the number of growing-season days the plants experienced before first flowering. Our results and the threshold hypothesis suggest that the canalization effect primarily reflects whether plants flowered in the first growing season, and therefore this approach captures both the variance between and within seasons. In addition to flowering time, the *nFT* locus also influenced the (co)variance of ‘leaf number when flowering’, a well-defined quantitative trait often used as an indicator of the reproductive phenology. We therefore think these phenological traits reflect important underlying biological processes.

### *nFT* controls multivariate trait covariation

The covariance structure among multiple traits (**G** matrix) indicates the magnitude of potential genetic constraints and can have profound effect on the magnitude and direction of trait evolution under selection [3], [58]–[60]. While many methodological and empirical studies have compared the **G** matrix evolution among evolutionary lineages [e.g., 3], only a few studies have investigated the genetic mechanism or identified the genomic regions responsible for this **G** matrix difference. In *Arabidopsis*, Stinchcombe *et al.* [61] found that two alleles of the *ERECTA* gene confer different structure of the **G** matrix among four traits. In mice, Wolf *et al.* [62] also identified significant epistatic pleiotropy effects of QTL on the covariance between traits. Both examples investigated the change of covariance structure caused by candidate loci, and to our knowledge, our study may be one of the first attempts at genome-wide mapping of QTL altering the covariance structure among multiple traits.

In addition to canalization in single traits, we have identified *nFT* as the major QTL altering the genetic covariance structure in both multivariate trait combinations: 1) between different traits in the same environment (indicating the magnitude of genetic constraint), and 2) the same trait between different environments (indicating the magnitude of GxE, the genotype-by-environment interaction component of plasticity). This supports the idea that genetic constraint and plasticity are related concepts [63] and can be connected by trait- or environment-dependent canalization: When one trait is canalized but another is not, the magnitudes of genetic constraint or GxE are altered [55]. For example, considering the relationship between flowering time and leaf number at flowering under 16 hour days, 25°C, 4 week vernalization, the *nFT* Colorado genotypes canalize flowering time, but there is no *nFT* canalization effect for leaf number (Figure 5). This changes the orientation of covariance structure by ∼35°. For GxE, the *nFT* Montana genotype canalizes flowering time in 12 hour days, 18°C, 4 week vernalization, and the Colorado genotype canalizes flowering time in 16 hour days, 25°C, 4 week vernalization. This leads to a significant difference in the orientation of the **G** matrices for the two *nFT* genotypes (∼59°, Figure 6, row 1, column 5). Therefore, it is helpful to view the multi-trait covariance structure as a multivariate extension of single-trait variance, and our result supports the idea that the change of magnitude in genetic constraint or GxE may be a consequence of trait- or environment-dependent canalization effects [64]. Our results also suggest that, in a threshold-like gene regulation system, the change of trait genetic covariance structure may be achieved simply by the shift of the gene activation threshold (Figures 3 and S9).

As described in the Introduction, plasticity consists of *V_E_* and *V_GxE_*. In this study we showed that the *nFT* QTL altered the magnitude of *V_GxE_*. It is also possible that genetic mechanisms altering *V_E_* exist. With this genetic mechanism (here termed macro-environmental canalization, as opposed to micro-environmental canalization which concerns *V_e_*, the variance from stochastic noise), one genotype of the QTL may exhibit similar phenotypes across diverse environments while the other genotype has varying phenotypes. While we did not specifically map for this macro-environmental canalization, the genetic mechanism altering *V_E_* may also be realized from the threshold hypothesis (Figure 3): consider the flowering time under two hypothetical environments, one strongly promotes flowering (‘ENV1’ hereafter: all genomic backgrounds generate signal above the Colorado threshold – all dots are white in Figure 3) and the other has effect between ‘slightly inhibiting’ and ‘strongly inhibiting’ (‘ENV2’ hereafter: all genomic backgrounds generate input signal above Montana but below the Colorado threshold – all dots are grey in Figure 3). Given the low threshold of the *FT* Montana genotype, all genomic backgrounds flower in both environments, and the *V_E_* for the Montana genotype is low. For the Colorado genotype, however, all genomic backgrounds flower under ENV1 but none flowers in ENV2, and *V_E_* is large for the Colorado genotype. Therefore, although in terms of upstream input signals the *V_E_* stays the same regardless of *FT* genotypes, for flowering time *FT* may control macro-environmental canalization due to the threshold-type reaction to upstream signals. This also suggests that environment-dependent genetic canalization, macro-environmental canalization, and the alteration of the magnitude in GxE may represent different viewpoints of the same concept.

### HIF experiment and the effect of *nFT*

Results from the HIF experiment show strong *nFT* by genomic background epistatic effects on phenological traits and on expression of the *FT* locus, demonstrating: 1) *nFT*'s role as an epistatic modifier of other flowering genes and 2) the effect of other flowering genes (different HIF genomic background) on gene expression of the *FT* locus itself, a network hub integrating signals from upstream genes in the flowering time pathway. These observations are consistent with the ‘threshold hypothesis’ illustrating how flowering pathway function can generate epistasis between *FT* and other flowering genes (Figure 3) and also echo studies with gene by genomic background epistatic effects in the flowering time pathway of *Arabidopsis* [65]. Unlike the strong and direct epistatic relationship between *Arabidopsis* flowering genes *FRIGIDA* (*FRI*) and *FLOWERING LOCUS C* (*FLC*) [37], [66], *FT* responds to the combined effect of multiple upstream pathways, and the one-to-one epistasis between *nFT* and individual flowering time loci may be too weak to be detected by our previous [25] or current study (Table S3). Our novel algorithm to map (co)variance QTL therefore serves as a valuable alternative to standard pairwise searches for epistasis, paralleling recent developments in human genetics [15], [67]. In the flowering time of *B. stricta*, this epistatic relationship between *nFT* and genomic background has been supported by our HIF experiment.

In this study we employed a linkage mapping approach to map (co)variance QTL, and the issue of sample size and statistical power may be a limitation [68]–[70]. We recognize that the experimental design may not have sufficient power to detect all QTL, especially those with minor effects. This limitation, however, does not affect the significant functional variation at *nFT*. Although it might be possible that the *nFT* QTL's effect on trait mean, variance, and covariance structure are effects of several closely linked genes, our threshold hypothesis and following HIF experiments both suggest that the *FT* gene may exhibit these pleiotropic effects: being a floral signal integrator, the two *FT* alleles may influence trait means due to different thresholds for activation by upstream signals (which predicts and is supported by our results that the two alleles vary in gene regulation patterns instead of amino acid substitutions). Such threshold differences may interact with various genomic backgrounds or environmental stimuli and thus alter the pattern of trait (co)variation (see Results and Figure 3). Further, it is not uncommon that genes or QTL can simultaneously control trait means and (co)variances, as previous studies mapping canalization loci have identified QTL or genes known to control trait means [7]–[10], and a recent study has shown strong genetic correlation between developmental instability (environmental canalization) and phenotypic plasticity [71]. Taken together, these results suggest that, at least in traits with major signal integrators such as flowering time, the control of trait means, (co)variances, and genotype-by-environment interaction may have a similar genetic basis.

## Materials and Methods

### Recombinant inbred line (RIL) data

All RIL data were obtained from our previous study [25]. Briefly, a cross was made between one genotype from Montana and one from Colorado [72], and F6 RIL were generated through self-pollination and single seed decent. From each family, one F6 individual was genotyped at 164 polymorphic molecular markers, with an average spacing of 5.5 cM between neighboring markers. The F6 RILs were predominantly homozygous (95.9%). Heterozygous genotype calls in any marker of any family were treated as missing data.

In the previous study, we measured flowering time and leaf number at flowering (*N* = 5 individuals/RIL/treatment and *N* = 35 individuals/parental line/treatment) in six distinct environments, composed of two vernalization lengths (four or six weeks) at 4°C and three growth conditions (12 hour days 18°C, 16 hour days 18°C, and 16 hour days 25°C). In this study, we analyze family mean trait values for the 178 RIL and 2 parental lines obtained from the previous study [25]. The growth chamber experiments consisted of two growing seasons. Individuals that had not flowered within 180 days after the first vernalization were subject to another 6-week vernalization. In addition, during the second growing season, plants from the 16 hour days 25°C chambers were moved to 16 hour days 18°C [25]. Two traits from each growing condition were used in this study: flowering time and leaf number at the time of first flowering. Flowering time is defined as the number of elapsed days since the end of the first vernalization, and the 6-week period of the second vernalization was excluded from the flowering time estimation. All traits were standardized to a mean of zero and standard deviation of one before further analysis.

### QTL controlling variance of single traits

For each genetic marker, we used the Brown-Forsythe test, a modification of Levene's test based on median, to estimate the difference in trait variance between the Colorado homozygote and the Montana homozygote at markers across the genome. This approach is similar to Shen et al. [8] and can detect QTL responsible for genetic canalization. We determined the statistical significance by the genome-wide permutation method of Churchill and Doerge [73]. One thousand permuted datasets were generated by randomizing trait values with respect to marker genotypes. The marker-trait relationship was randomized, but the genotype vector and the trait vector for each individual were not altered. From each permuted data set, the Brown-Forsythe statistic was calculated at each genetic marker, and the genome-wide maximum Brown-Forsythe value was recorded, providing a genome-wide null probability distribution. The *P-value* of the Brown-Forsythe statistics for each marker in the observed data was obtained by comparing this value to the null distribution of Brown-Forsythe values from the 1,000 randomized datasets. Our genome-wide permutation procedure provides a straightforward control for multiple tests across all markers and is also robust to violations of the assumption of multivariate normality. The estimation of variance and covariance, however, may be limited by small sample size, perhaps resulting from missing data or segregation distortion. To prevent possible bias, we therefore excluded six markers with minor allele frequency less than 0.33. All computations were performed in R (http://www.r-project.org/) using scripts available upon request from CL.

Considering the effect of a QTL, the total trait variation (*V_p_*) can be decomposed into:
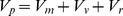
where *V_m_* is the variation explained by the difference in mean of the two homozygous genotypes, *V_v_* is the variation explained by the difference in variance of the two genotypes, and *V_r_* is residual variance arising from other sources [8]. For each significant QTL, we calculated the proportion of variation explained by *V_m_* and *V_v_*, following previously published equations designed for populations with two homozygous genotypes in each SNP [8]:
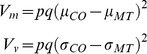
where *p* and *q* are the genotype frequencies of the Colorado and Montana homozygotes, respectively. *μ_CO_* and *μ_MT_* are the mean, and *σ_CO_* and *σ_MT_* represent the standard deviation of the Colorado and Montana homozygotes.

### QTL controlling the structure of covariance matrix among multiple traits

Here we aim to map QTL altering the covariance structure of three groups of traits: 1) flowering time and plant size at flowering (number of leaves) in all six environments (**G** matrix with 12 traits); 2) flowering time in all environments (**G** matrix of six traits); 3) plant leaf number at flowering in all environments (**G** matrix with six traits). We further mapped QTL changing the covariance structure between flowering time and plant size at flowering separately for each environment (representing the magnitude of genetic constraint) and between the same trait in pairs of different environments (representing the magnitude of the genotype-by-environment interaction component of plasticity, GxE). Among the multiple ways to model plasticity (reviewed in [20], [74], here following Falconer [21]), we treat the same trait in distinct environments as separate traits and model their covariance structure. We choose this definition because this view generalizes both GxE and genetic constraint into the relationship among traits, allowing the use of established methods for **G** matrix comparisons.

For each molecular marker, we separated the data into two groups of homozygous genotypes. Two separate (co)variance matrices (**G** matrices) were estimated, and we assessed the QTL effect by comparing the **G** matrices via three methods: 1) Box's M statistics; 2) the angle between G_max_; 3) the Krzanowski index. Statistical significance is determined by the genome-wide permutation algorithm described above. We acknowledge that the covariance matrix estimated from family means may not be identical to the genetic covariance matrix estimated from individual-level mixed-model MANOVA. This simplification, however, was necessary to ensure computational feasibility since the **G** matrices needed to be calculated twice (one for each homozygous genotype of a marker) for ∼160 markers for each of the 1,000 permuted data sets.

Box's M statistic [31] compares the difference between the trace of multiple covariance matrices and the trace of their pooled covariance matrix:
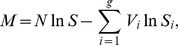
where *g* is the number of matrices to be compared (two in our case), *V_i_* is the degrees of freedom, and *S_i_* is the trace of the *i*-th matrix. *N*, the overall degrees of freedom, is the sum of all *V_i_* values. The trace, *S*, of the pooled covariance matrix is:
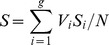
Since the trace of a covariance matrix is the sum of its diagonal elements and is equal to the sum of eigenvalues from its principal components, the Box's M value could be interpreted as the difference between the multivariate volumes occupied by different covariance matrices. In our case, the Box's M method compares the overall size of **G** matrices from the two genotypes at each genetic marker. Traditionally the significance is determined by an F-test and is sensitive to deviations from multivariate normality, but our genome-wide permutation procedure alleviates this parametric distributional requirement.

Two covariance matrices could differ not only in size but also in their orientation. We used two methods to compare the orientation between **G** matrices [32], [75]. To estimate the radian angle between the respective G_max_ (first principal component), we first calculated the angle between the first eigenvector, **u** and **v**, of the two **G** matrices respectively:
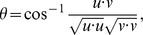
where the dot symbol calculates the dot product between vectors. Since eigenvectors are directional, *θ* may be larger than *π*/2 (90 degrees, the maximum possible angular difference between two non-directional axes). Therefore if *θ* is larger than *π*/2, the radian angle between G_max_ is calculated as *π* - *θ*, otherwise the angle equals *θ*. This method estimates the angular difference between the respective axes with most variation in each **G** matrix.

The G_max_ method, however, has a caveat that when **G** matrices have many dimensions (traits), other principal components may carry substantial amounts of variation, and comparing G_max_ may not be sufficient [32]. Therefore, we employed the method of Krzanowski [33], which has also been used in recent studies [32]. In brief, this method compares the *k*-dimensional subspace between two **G** matrices, where *k* is less than or equal to half of the dimension of the original **G** matrix. For example, in a data set with 10 traits, we estimated the degree of similarity between two subspaces formed by the first five eigenvectors of two **G** matrices. Similar to the G_max_ method above, each eigenvector **w** that will be used in the analysis was first standardized by the square root of its dot product:
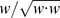
The ‘matrix of similarity’ (**S**) then is calculated as:
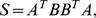
where **A** and **B** are matrices containing the first five standardized eigenvectors of the two respective **G** matrices, and superscript T denotes matrix transpose. As in other studies [32], we used the sum of eigenvalues of this **S** matrix (the Krzanowski index) as a measure of overall similarity between the two subspaces. This index ranges from 0 to 5 in our example of 10 traits, with 0 signifying non-overlap and 5 indicating total overlap between subspaces. For our purpose of mapping QTL whose different genotypes confer the most dissimilar **G** matrices, we compared the negative Krzanowski index of each marker to the 1,000 maximum negative Krzanowski indexes from permutation.

In summary, while the G_max_ and Krzanowski methods compare the orientation of linear relationship among traits, Box's M tests the dispersion of points from this linear relationship. All mapping algorithms were written in R (http://www.r-project.org/). When only two traits are involved, the angle between G_max_ captures all the difference in orientation between **G** matrices, and therefore the Krzanowski method is not necessary.

### Heterogeneous inbred family phenotypic experiment

To test the existence of epistasis as predicted by the threshold hypothesis, we performed analysis of variance for the interaction effect between *nFT* and other flowering time QTL identified in the same growth chambers from our previous study [25]. The epistatic effects of other QTL were tested separately, using flowering time as response and *nFT*, the other QTL, and their interaction as fixed-effect predictor variables in each model.

We generated four heterogeneous inbred families (HIFs, Figure S8) to test the epistatic effect between nFT and genomic background (the cumulative effect of other genes in the genome). Based on the genotype data in the F6 generation [25] we identified four F5 parents heterozygous at *nFT* and mostly homozygous at other markers. From each F5 parent we planted approximately 250 seeds, which are self-full siblings of the original genotyped F6 individual in Anderson *et al.* [25] (*N* = 1097 plants total from four F5 parents). We genotyped the microsatellite marker C02 [∼5 cM from the FT gene,25] in all plants and collected seeds from those that were homozygous at C02. Seeds from the same F6 individual (a ‘family’ hereafter) have virtually identical genomic composition. All plants within the same HIF are nearly identical in other genomic regions but segregate for two *nFT* homozygous genotypes. With four HIFs that are different in genomic background, the interaction between *nFT* genotype and HIF (genomic background) provides a statistical test for epistatic effects on phenology.

The HIF experiment was conducted in the Duke University Greenhouse rather than in multiple growth chamber environments as in the RIL experiment. To provide independent replication for each homozygous *nFT* genotype, we selected at least 20 homozygous families from each HIF (24 families from HIF 3A; 22 families from HIF 89A; 23 families from HIF 98A; and 20 families from HIF 105A), for a total of 89 families. In November 2011 we place 10–15 seeds from each of the 89 families on moist filter paper in petri dishes in dark conditions at ambient temperature for 3 weeks until germination. As in other *B. stricta* greenhouse experiments [76], seedlings were then planted in Ray Leach SC10 ‘Cone-tainers’ (21 cm in depth and 3.8 cm in diameter, Stuewe & Sons Inc., Tangent, OR, USA), with the lower 80% of each Cone-tainer filled with Fafard 4P Mix soil (Conrad Fafard, Agawam, MA, USA) and top 20% with Sunshine MVP soil (Sun Gro Horticulture, Vancouver, BC, Canada). Greenhouse conditions were as follows: 16-hour days (6 AM to 10 PM), diurnal temperature of 18–21°C, and nocturnal temperature of 13–16°C. We used a random number generator to assign seedlings to distinct positions in 9 blocks, each containing 91–96 plants. Each block included individuals from all HIFs and most families from each HIF (In some cases, a family did not have enough siblings to be represented in each block). The blocks were rotated around a greenhouse bench once a week to minimize the effects of environmental gradients in the greenhouse.

In January 2012, all rosettes were vernalized at 4°C for 8 weeks. Plants were removed from vernalization on 29 February 2012, at which point we monitored them 7 days/week and recorded the date of first flowering as well as the number of leaves and plant height at first flowering. By April 23, 2012, we had collected phenological data from 8–10 full siblings per family (*N* = 785 F7 individuals flowered successfully). No individuals flowered after that date. Relevant data are available in Dataset S1.

Statistical analysis was performed with REML mixed-model ANOVA (Proc Mixed, SAS 9.3, SAS, Cary, NC). We first conducted a multivariate ANOVA (MANOVA) to address how the three response variables (day of first flowering, plant height and number of leaves at flowering) varied with HIF (3A/89A/98A/105A), *nFT* genotype (Montana/Colorado homozygote), and *nFT* by HIF interaction (all are fixed effects). We incorporated ‘family’ (nested within *nFT* homozygote, cross-classified with HIF) and block as random effects. We then conducted univariate ANOVA for each response variable with the same statistical model.

### *FT* gene expression in HIF

The contrasting canalization effect of the *nFT* locus in different environments suggests a three way interaction of the major QTL (*nFT*) by genomic background (the combination of other flowering-related genes) by environment conditions, and the interaction between *nFT* and genomic background on phenological traits is tested in the HIF experiment. The *nFT* locus contains the ortholog of the *FT* gene in *Arabidopsis* (AT1G65480). *FT* serves as a major hub for integrating upstream signals of flowering, and its expression often correlates with the onset of flowering [30]. If the variation in the *FT* gene in *Boechera stricta* is responsible for the differential canalization effect of the *nFT* QTL in our Montana by Colorado cross, its expression pattern should vary depending on the *nFT* genotype and genomic background. We therefore test the expression pattern of *FT* in the same HIF experimental design.

Two HIFs (HIF 89A and HIF 98A) were used in this experiment. Within each HIF we obtained five families from each homozygous *nFT* genotype for a total of 20 families. Forty experimental plants (two individuals from each family) were completely randomized, and all planting procedures and greenhouse environmental settings were as above. Rosettes were grown in the Duke greenhouse for 12 weeks and stratified at 4°C for 8 weeks. In *Arabidopsis*, *FT* mainly expresses in leaves, where protein translation happens, and the proteins are transferred to floral meristems [77]. We therefore collected one young leaf from each plant four weeks after vernalization ended. *FT* in *Arabidopsis* exhibits circadian rhythm in gene expression, and under 16-hour days, its maximum expression is in the end of daytime [78]–[80]. We therefore collected leaves from all 40 experimental plants around 10 pm, when the 16-hour Duke greenhouse days end. Leaves were immediately flash frozen in liquid nitrogen and stored at −80°C. RNA was extracted with Sigma Spectrum Plant Total RNA Kit, and cDNA was synthesized with Thermo Scientific DyNAmo cDNA Synthesis Kit. Two samples failed during the RNA extraction and cDNA synthesis steps, leaving 38 samples in total.

Our partial genomic sequencing shows that there may be more than one *FT* gene copy in *Boechera stricta* (Joint Genome Institute and Mitchell-Olds lab, unpublished). Therefore, we cloned and sequenced *FT* full-length coding sequences from both parents. Only one copy is expressed, and both parents have the same expressing copy with identical coding region sequences (KJ576855 and KJ576856 in GenBank, where the Montana genotype is denoted as ‘LTM’ and Colorado genotype as ‘SAD12’). All primer sequences are available in Table S4. *FT* gene expression was measured by quantitative PCR (qPCR) with Thermo Scientific DyNAmo SYBR Green qPCR Kits. Following previous experiments [81], the *ACTIN2* gene (*ACT2*) is used as reference gene, and *FT* expression level for each of the 38 samples was calculated as:
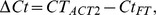
where *Ct_ACT2_* is the *Ct* value in qPCR of the reference gene *ACTIN2*, and *Ct_FT_* is the *Ct* value of *FT*. Since within each sample the *Ct* value of *FT* is always larger (i.e., the signal is lower) than *ACT2*, Δ*Ct* is always negative, and larger Δ*Ct* represents higher *FT* gene expression. The relative qPCR signal of *FT* to *ACT2* can be calculated as 2^Δ*Ct*^. This 2^Δ*Ct*^ value, however, has a skewed distribution among samples. Since log transformation of 2^Δ*Ct*^ yields a value that is proportional to Δ*Ct*, we used the original Δ*Ct* as the response variable for statistical analysis. Relevant data are available in Dataset S2.

Statistical analysis was performed as in the HIF phenotypic experiment, where *nFT* genotype, HIF, and *nFT* by HIF interaction were treated as fixed effects, and family was treated as a random effect nested within *nFT* and HIF. All 40 plants were grown in the same block, so no block effect exists for this experiment. To further test if *FT* expression in *Boechera stricta* is related with flowering, we recorded whether each of the 40 experimental plants had visible flowering buds during the time of leaf-tissue collection. The analysis incorporates Δ*Ct* as the response variable, and the phenological indicator ‘whether a plant has visible bud’ as a fixed-effect categorical predictor, and family as random-effect predictor variable.

## Supporting Information

Figure S1 QTL controlling the variance of phenological traits in growth chamber environments. Each row represents a trait in one environment. The six upper rows are flowering time, and the six lower rows are leaf number when flowering in six different environments. Texts beside each row represent the environment and trait. For example, ‘12 H, 18 C, 4 W, FT’ refers to flowering time under 12 hour days, 18 degree C, and 4 weeks of vernalization, and ‘16 H, 25 C, 6W, LN’ refers to leaf number under 16 hour days, 25 degree C, and 6 weeks of vernalization. Each column represents a genetic marker on the linkage map, and chromosomes are separated by vertical white lines. Black cells represent markers significantly controlling the variance of a trait, whereas dark grey cells are non-significant markers. Light grey cells are markers that are excluded due to segregation distortion (see main text).
(PDF)

Figure S2 Flowering time distributions of families with the Montana (red bars) or Colorado (blue bars) homozygous genotypes of two QTL (BST031941 and Bst004238) in the environment with 16 hour days, 25°C, and 4 weeks of vernalization. Above each graph, horizontal bars denote the mean plus or minus one standard deviation for each allele, numbers on the left denote percent of total variation explained by the difference in variance of the two alleles, and asterisks on the right denote genome-wide significance of the difference in variance. * *P*< = 0.05, ** *P*< = 0.01, *** *P*< = 0.001.
(PDF)

Figure S3 ‘Leaf number when flowering’ distributions of families with the Montana (red bars) or Colorado (blue bars) homozygous genotypes of *nFT* locus in six environments. Panels in a column have the same ambient environment: first column – 12 hour days 18°C, second column – 16 hour days 18°C, third column – 16 hour days 25°C. Panels in a row have the same vernalization treatment: first row – 4 week vernalization, second row – 6 week vernalization. Vertical dashed lines (180 days) separate the two growing seasons in each environment. Above each graph, horizontal bars denote the mean plus or minus one standard deviation for each allele, numbers on the left denote percent of total variation explained by the difference in variance of the two alleles, and asterisks on the right denote genome-wide significance of the difference in variance. * *P*< = 0.05, ** *P*< = 0.01, *** *P*< = 0.001.
(PDF)

Figure S4 QTL controlling the covariance structure of phenological traits in growth chamber environments. Each row represents a statistical method on a group of traits across several environments. For example, ‘Box's M, all traits’ represents the results of Box's M method on flowering time and leaf number from all six environments (12 traits total), and ‘Krzanowski, leaf number’ represents results of Krzanowski method on leaf number in six environments. Each column represents a genetic marker on the linkage map, and chromosomes are separated by vertical white lines. Black cells represent markers significantly controlling the covariance of a group of traits, whereas dark grey cells are non-significant markers. Light grey cells are markers that are excluded due to segregation distortion (see main text).
(PDF)

Figure S5 The effect of QTL BST031941 on the structure of covariance matrix among standardized flowering time in all environments. Each dot represents the trait value of one recombinant inbred family, and an ellipse represents the 95% confidence region of the covariance matrix defined by an allele. Montana allele: black dots and solid ellipse. Colorado allele: white dots and dashed ellipse.
(PDF)

Figure S6 The effect of QTL BST031941 on the structure of covariance matrix between standardized flowering time and leaf number when flowering in each of the six environments. Asterisks on the upper right of each graph denote genome-wide significance for the Box's M method (ellipse size), and asterisks on the upper left of each graph denote significance for the G_max_ angle method (ellipse orientation). Montana allele: red dots and ellipse. Colorado allele: blue dots and ellipse. ** *P*< = 0.01, *** *P*< = 0.001.
(PDF)

Figure S7 The effect of QTL BST031941 on the plasticity of standardized phenological traits. Each graph shows the relationship of the same trait (above the diagonal – flowering time; below the diagonal – leaf number when flowering) between pairs of environments. Montana allele: red dots and ellipse. Colorado allele: blue dots and ellipse. In each graph, asterisks in the upper right denote genome-wide significance for the Box's M method (ellipse size), and asterisks on the upper left denote genome-wide significance for the G_max_ angle method (ellipse orientation). * *P*< = 0.05, ** *P*< = 0.01, *** *P*< = 0.001.
(PDF)

Figure S8 Generation of Heterogeneous Inbred Family (HIF) for the *nFT* locus. Shown are examples of two HIF. Each HIF was generated from one F5 parent that is almost homozygous across the genome but heterozygous for the *nFT* locus. The F5 plant is self-fertilized to generate many F6 plants, and all of them are genotyped for *nFT*. The F6 plants that are homozygous for *nFT* are self-fertilized to generate F7 plants for the experiments. All F7 siblings from the same F6 parent are nearly clones to each other, and all F7 plants within a HIF are almost identical in the genome but segregating for two homozygous *nFT* genotypes. Different HIF differ in genomic background, therefore allowing the test for *nFT* by genomic background interaction effect.
(PDF)

Figure S9 The threshold hypothesis is related with the sigmoid model of gene regulation. In a sigmoid model *y* = 1/(1+exp(*a* * (−*x*+*b*))), *x* is the upstream input signal from different genomic backgrounds or environments (horizontal axes in panel A, B, and C, which is identical to the vertical axis of Figure 3), *y* is the probability of FT expression (vertical axes in panel A and B), *a* determines the width of the log phase in the sigmoid curve, and *b* determines the *x* coordinates where the log phase is centered. The sigmoid model approximates the threshold hypothesis when the value of parameter *a* becomes larger, which makes the log phase narrower and finally converge at coordinate *b*, the threshold. The sigmoid function of the Colorado (panel A) and the Montana (panel B) genotypes of *FT* simply differ in their threshold, the value of parameter *b* and *b′* as marked by the vertical dashed blue and red lines. Panel C is similar to Figure 3.
(PDF)

Table S1 Pairwise genetic correlations (upper diagonal) and the *P* values (lower diagonal) between 12 phenological traits in this study.
(DOCX)

Table S2 Trait loadings on the first two principal components for all traits, flowering time traits, and leaf number traits shown in Figure 4.
(DOCX)

Table S3 Statistical tests of the interaction effect (epistasis) between *nFT* and other flowering-time QTL identified in a previous study.
(DOCX)

Table S4 Primer sequences used in this study.
(DOCX)

Dataset S1 Data for the greenhouse experiment for *nFT* by HIF effect on phenological traits. Block: Experimental blocks, as categorical random effect; Flowering_time: Days of first flowering after vernalization, as numeric response variable; Leaf_number_at_flowering: Leaf number when flowering, as numeric response variable; Height_at_flowering: Plant height at flowering (cm), as numeric response variable; nFT_genotype: The genotype at the C02 microsatellite locus near nFT QTL, as categorical fixed effect; HIF: HIF family, as categorical fixed effect; Family: The F6 individuals those experimental F7 plants were descended from, as categorical random effect nested within HIF and nFT_genotype.
(TXT)

Dataset S2 Data for the greenhouse experiment for *nFT* by HIF effect on *FT* gene expression. Plant_ID: ID of individual plants; HIF: HIF family, as categorical fixed effect; Family: The F6 individuals those experimental F7 plants were descended from, as categorical random effect nested within HIF and nFT_genotype; nFT_genotype: The genotype at the C02 microsatellite locus near nFT QTL, as categorical fixed effect; Visible_bud: Whether the individual plant has visible flowering buds at the time of tissue collection; delta_Ct: Ct value of *ACT2* minus *FT*.
(TXT)
