# Supplementary figures and images for: Unifying Genetic Canalization, Genetic Constraint, and Genotype-by-Environment Interaction: QTL by Genomic Background by Environment Interaction of Flowering Time in Boechera stricta
Source: PLoS Genet. 2014 Oct 23;10(10):e1004727. doi: 10.1371/journal.pgen.1004727 (PMC4207664; doi:10.1371/journal.pgen.1004727)

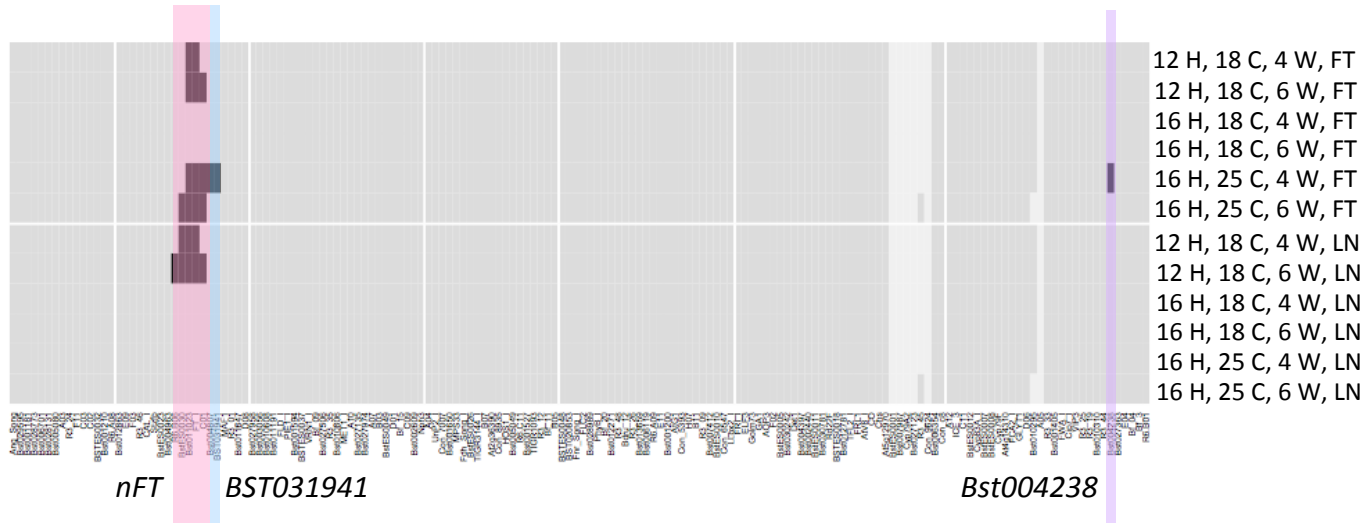

Supplement: Figure S1 — QTL controlling the variance of phenological traits in growth chamber environments. Each row represents a trait in one environment. The six upper rows are flowering time, and the six lower rows are leaf number when flowering in six different environments. Texts beside each row represent the environment and trait. For example, ‘12 H, 18 C, 4 W, FT’ refers to flowering time under 12 hour days, 18 degree C, and 4 weeks of vernalization, and ‘16 H, 25 C, 6W, LN’ refers to leaf number under 16 hour days, 25 degree C, and 6 weeks of vernalization. Each column represents a genetic marker on the linkage map, and chromosomes are separated by vertical white lines. Black cells represent markers significantly controlling the variance of a trait, whereas dark grey cells are non-significant markers. Light grey cells are markers that are excluded due to segregation distortion (see main text). (PDF) [file pgen.1004727.s001.pdf]

BST031941

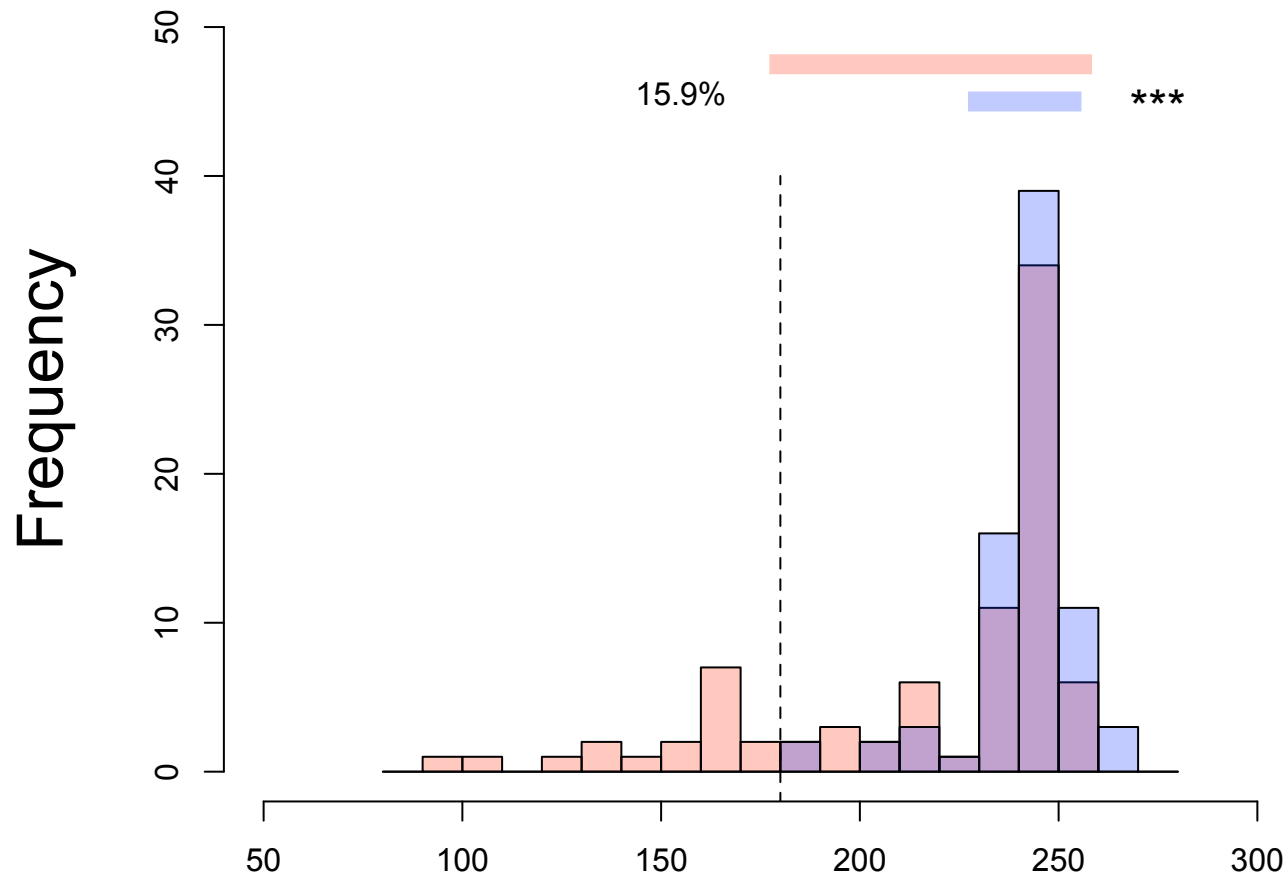

Bst004238

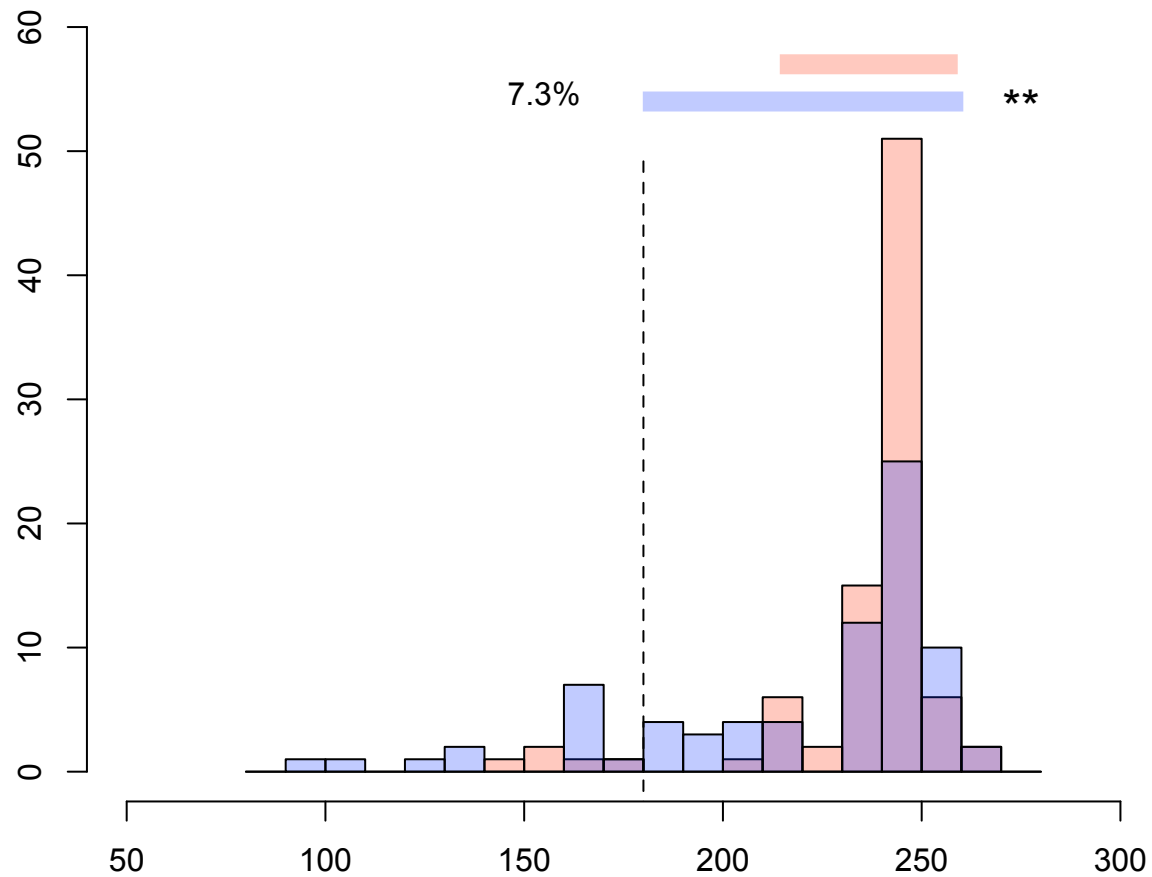

Supplement: Figure S2 — Flowering time distributions of families with the Montana (red bars) or Colorado (blue bars) homozygous genotypes of two QTL (BST031941 and Bst004238) in the environment with 16 hour days, 25°C, and 4 weeks of vernalization. Above each graph, horizontal bars denote the mean plus or minus one standard deviation for each allele, numbers on the left denote percent of total variation explained by the difference in variance of the two alleles, and asterisks on the right denote genome-wide significance of the difference in variance. * P< = 0.05, ** P< = 0.01, *** P< = 0.001. (PDF) [file pgen.1004727.s002.pdf]

# BST031941

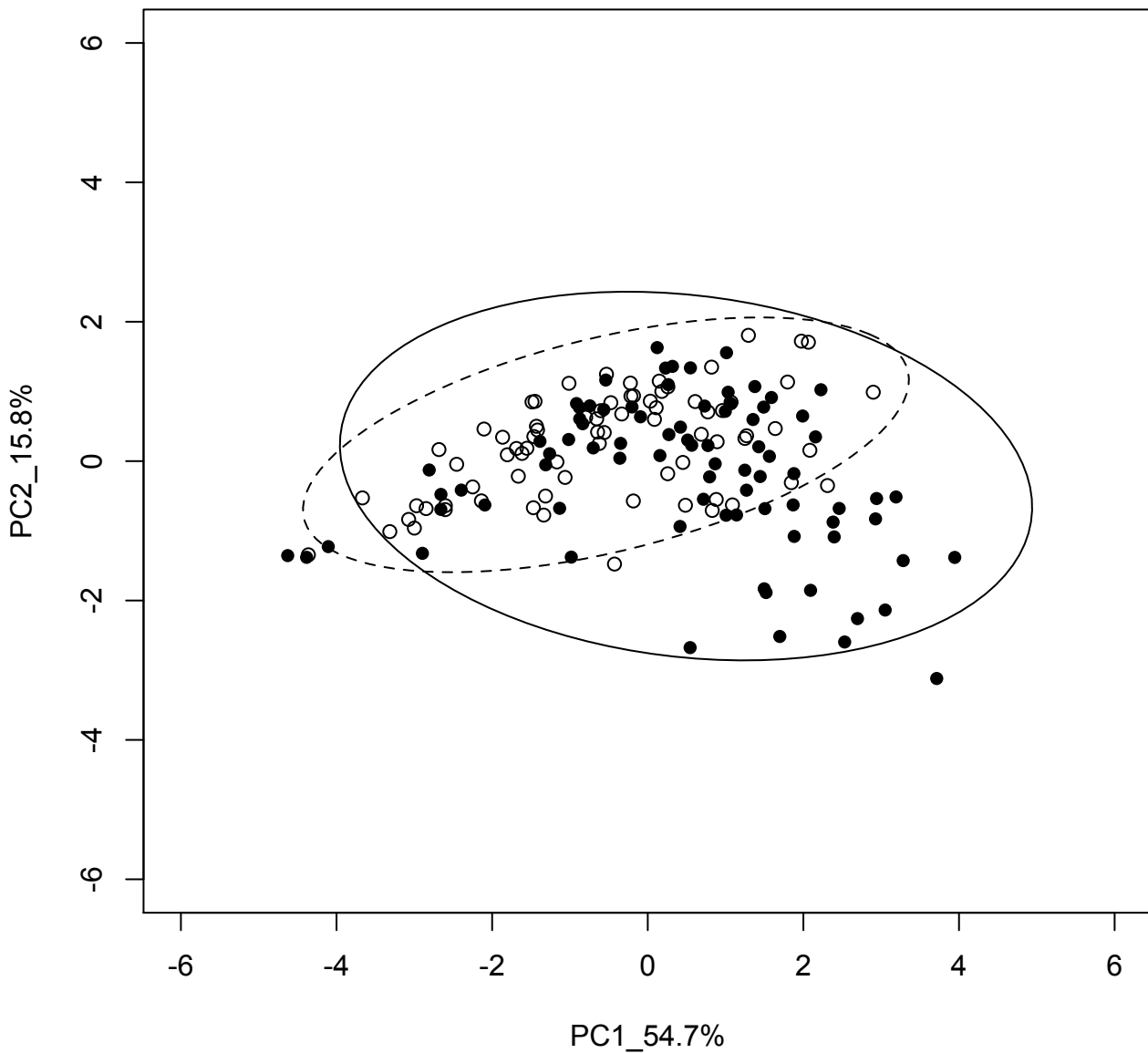

Supplement: Figure S5 — The effect of QTL BST031941 on the structure of covariance matrix among standardized flowering time in all environments. Each dot represents the trait value of one recombinant inbred family, and an ellipse represents the 95% confidence region of the covariance matrix defined by an allele. Montana allele: black dots and solid ellipse. Colorado allele: white dots and dashed ellipse. (PDF) [file pgen.1004727.s005.pdf]

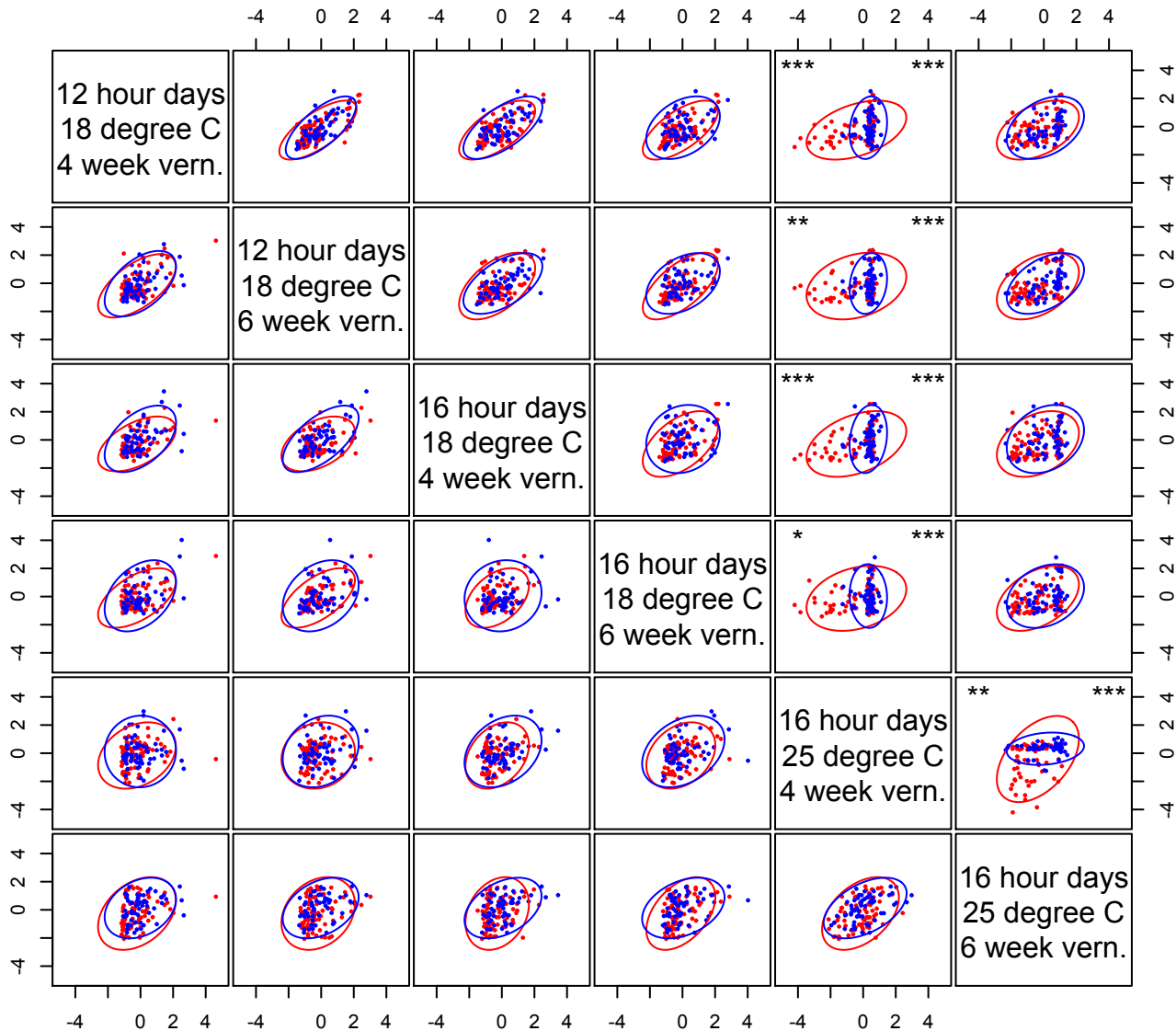

Supplement: Figure S7 — The effect of QTL BST031941 on the plasticity of standardized phenological traits. Each graph shows the relationship of the same trait (above the diagonal – flowering time; below the diagonal – leaf number when flowering) between pairs of environments. Montana allele: red dots and ellipse. Colorado allele: blue dots and ellipse. In each graph, asterisks in the upper right denote genome-wide significance for the Box's M method (ellipse size), and asterisks on the upper left denote genome-wide significance for the Gmax angle method (ellipse orientation). * P< = 0.05, ** P< = 0.01, *** P< = 0.001. (PDF) [file pgen.1004727.s007.pdf]

HIF A (F5)

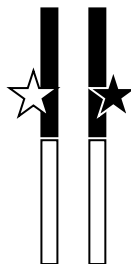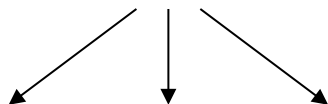

Families (F6)

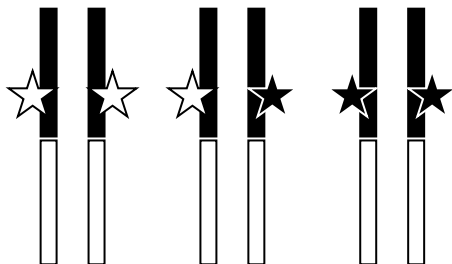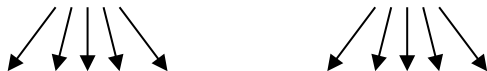

Experimental plants (F7)

HIF B (F5)

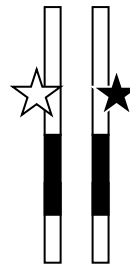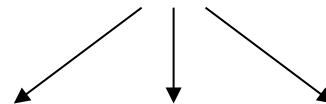

Families (F6)

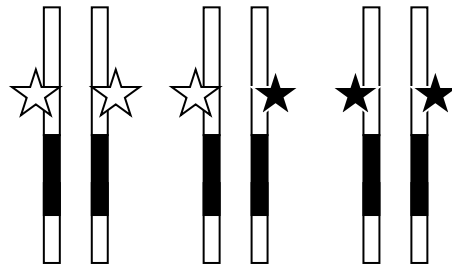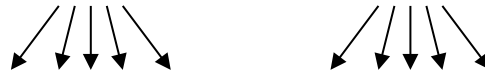

Experimental plants (F7)

Supplement: Figure S8 — Generation of Heterogeneous Inbred Family (HIF) for the nFT locus. Shown are examples of two HIF. Each HIF was generated from one F5 parent that is almost homozygous across the genome but heterozygous for the nFT locus. The F5 plant is self-fertilized to generate many F6 plants, and all of them are genotyped for nFT. The F6 plants that are homozygous for nFT are self-fertilized to generate F7 plants for the experiments. All F7 siblings from the same F6 parent are nearly clones to each other, and all F7 plants within a HIF are almost identical in the genome but segregating for two homozygous nFT genotypes. Different HIF differ in genomic background, therefore allowing the test for nFT by genomic background interaction effect. (PDF) [file pgen.1004727.s008.pdf]
