# Supplementary material for: Unifying Genetic Canalization, Genetic Constraint, and Genotype-by-Environment Interaction: QTL by Genomic Background by Environment Interaction of Flowering Time in Boechera stricta
Source: PLoS Genet. 2014 Oct 23;10(10):e1004727. doi: 10.1371/journal.pgen.1004727 (PMC4207664; doi:10.1371/journal.pgen.1004727)
Supplement: Figure S3 — ‘Leaf number when flowering’ distributions of families with the Montana (red bars) or Colorado (blue bars) homozygous genotypes of nFT locus in six environments. Panels in a column have the same ambient environment: first column – 12 hour days 18°C, second column – 16 hour days 18°C, third column – 16 hour days 25°C. Panels in a row have the same vernalization treatment: first row – 4 week vernalization, second row – 6 week vernalization. Vertical dashed lines (180 days) separate the two growing seasons in each environment. Above each graph, horizontal bars denote the mean plus or minus one standard deviation for each allele, numbers on the left denote percent of total variation explained by the difference in variance of the two alleles, and asterisks on the right denote genome-wide significance of the difference in variance. * P< = 0.05, ** P< = 0.01, *** P< = 0.001. (PDF) [file pgen.1004727.s003.pdf]

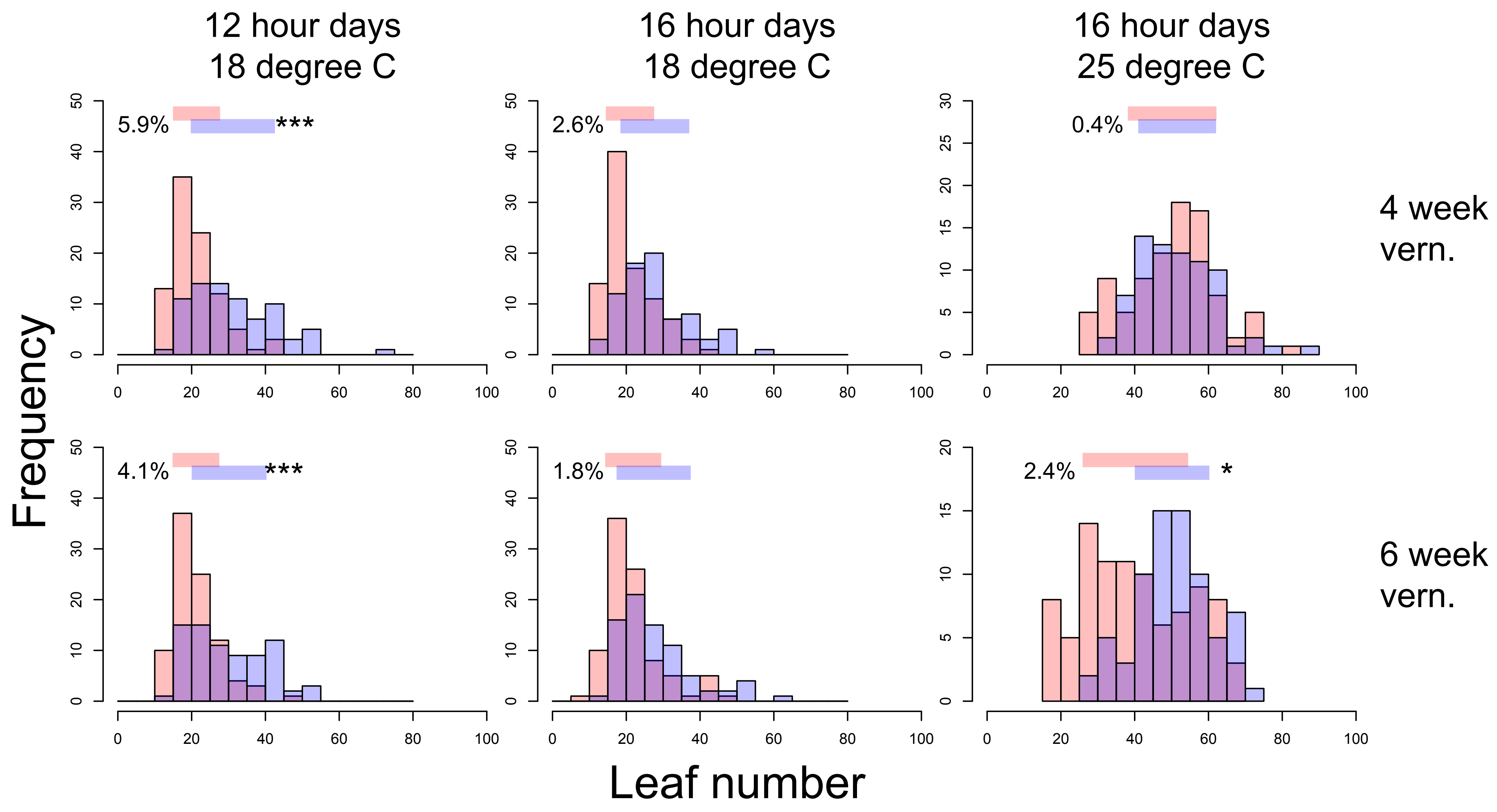

12 hour days
18 degree C
16 hour days
18 degree C
16 hour days
25 degree C
4 week vern.
6 week vern.
5.9%
***
2.6%
0.4%
4.1%
***
1.8%
2.4%
*
Frequency
Leaf number
0
20
40
60
80
100
