# Supplementary material for: Unifying Genetic Canalization, Genetic Constraint, and Genotype-by-Environment Interaction: QTL by Genomic Background by Environment Interaction of Flowering Time in Boechera stricta
Source: PLoS Genet. 2014 Oct 23;10(10):e1004727. doi: 10.1371/journal.pgen.1004727 (PMC4207664; doi:10.1371/journal.pgen.1004727)
Supplement: Figure S6 — The effect of QTL BST031941 on the structure of covariance matrix between standardized flowering time and leaf number when flowering in each of the six environments. Asterisks on the upper right of each graph denote genome-wide significance for the Box's M method (ellipse size), and asterisks on the upper left of each graph denote significance for the Gmax angle method (ellipse orientation). Montana allele: red dots and ellipse. Colorado allele: blue dots and ellipse. ** P< = 0.01, *** P< = 0.001. (PDF) [file pgen.1004727.s006.pdf]

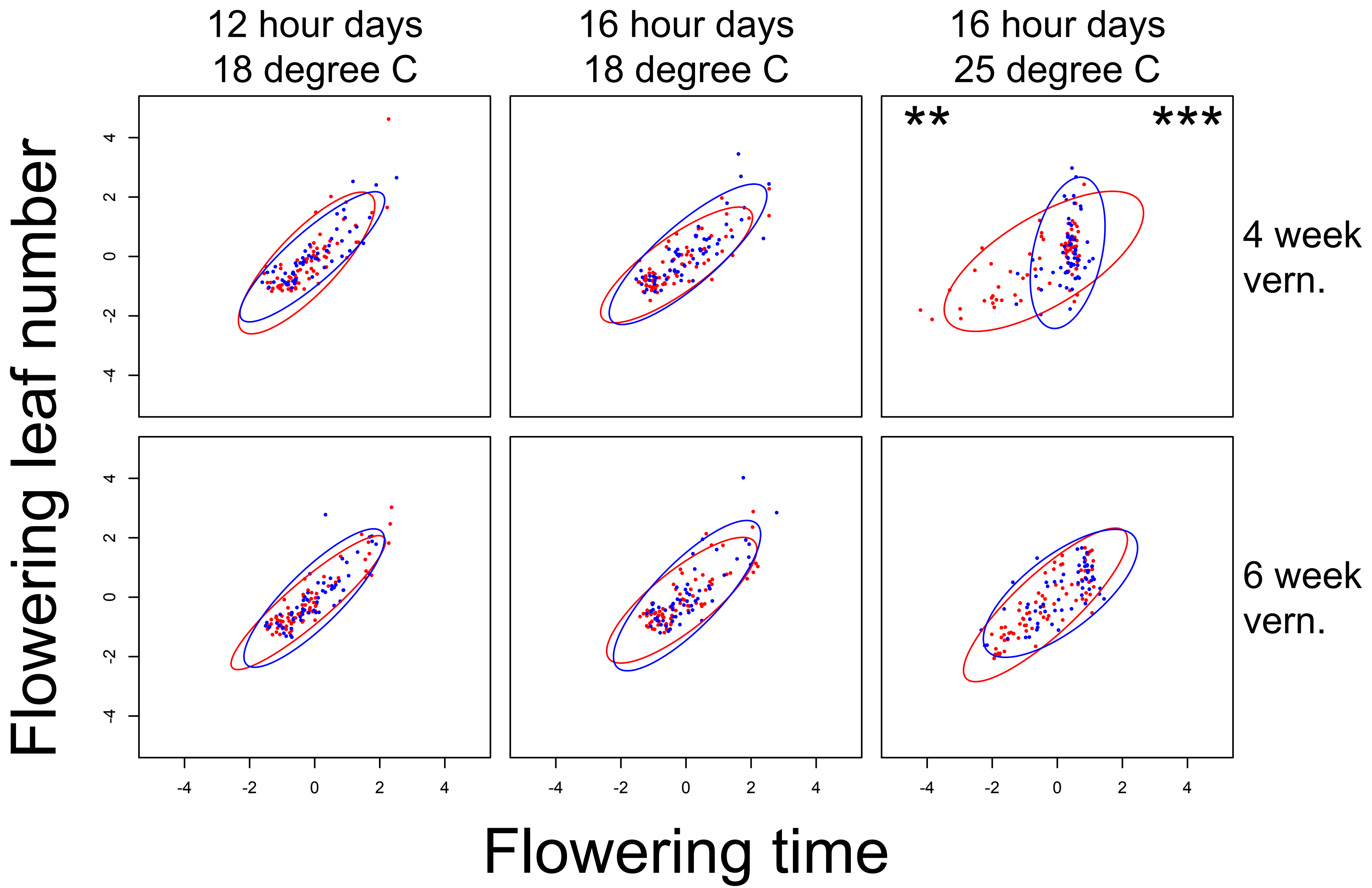

12 hour days
18 degree C
16 hour days
18 degree C
16 hour days
25 degree C
**
***
4 week
vern.
6 week
vern.
Flowering leaf number
Flowering time
-4
-2
0
2
4
