# Supplementary material for: Unifying Genetic Canalization, Genetic Constraint, and Genotype-by-Environment Interaction: QTL by Genomic Background by Environment Interaction of Flowering Time in Boechera stricta
Source: PLoS Genet. 2014 Oct 23;10(10):e1004727. doi: 10.1371/journal.pgen.1004727 (PMC4207664; doi:10.1371/journal.pgen.1004727)
Supplement: Figure S9 — The threshold hypothesis is related with the sigmoid model of gene regulation. In a sigmoid model y = 1/(1+exp(a * (−x+b))), x is the upstream input signal from different genomic backgrounds or environments (horizontal axes in panel A, B, and C, which is identical to the vertical axis of Figure 3), y is the probability of FT expression (vertical axes in panel A and B), a determines the width of the log phase in the sigmoid curve, and b determines the x coordinates where the log phase is centered. The sigmoid model approximates the threshold hypothesis when the value of parameter a becomes larger, which makes the log phase narrower and finally converge at coordinate b, the threshold. The sigmoid function of the Colorado (panel A) and the Montana (panel B) genotypes of FT simply differ in their threshold, the value of parameter b and b′ as marked by the vertical dashed blue and red lines. Panel C is similar to Figure 3. (PDF) [file pgen.1004727.s009.pdf]

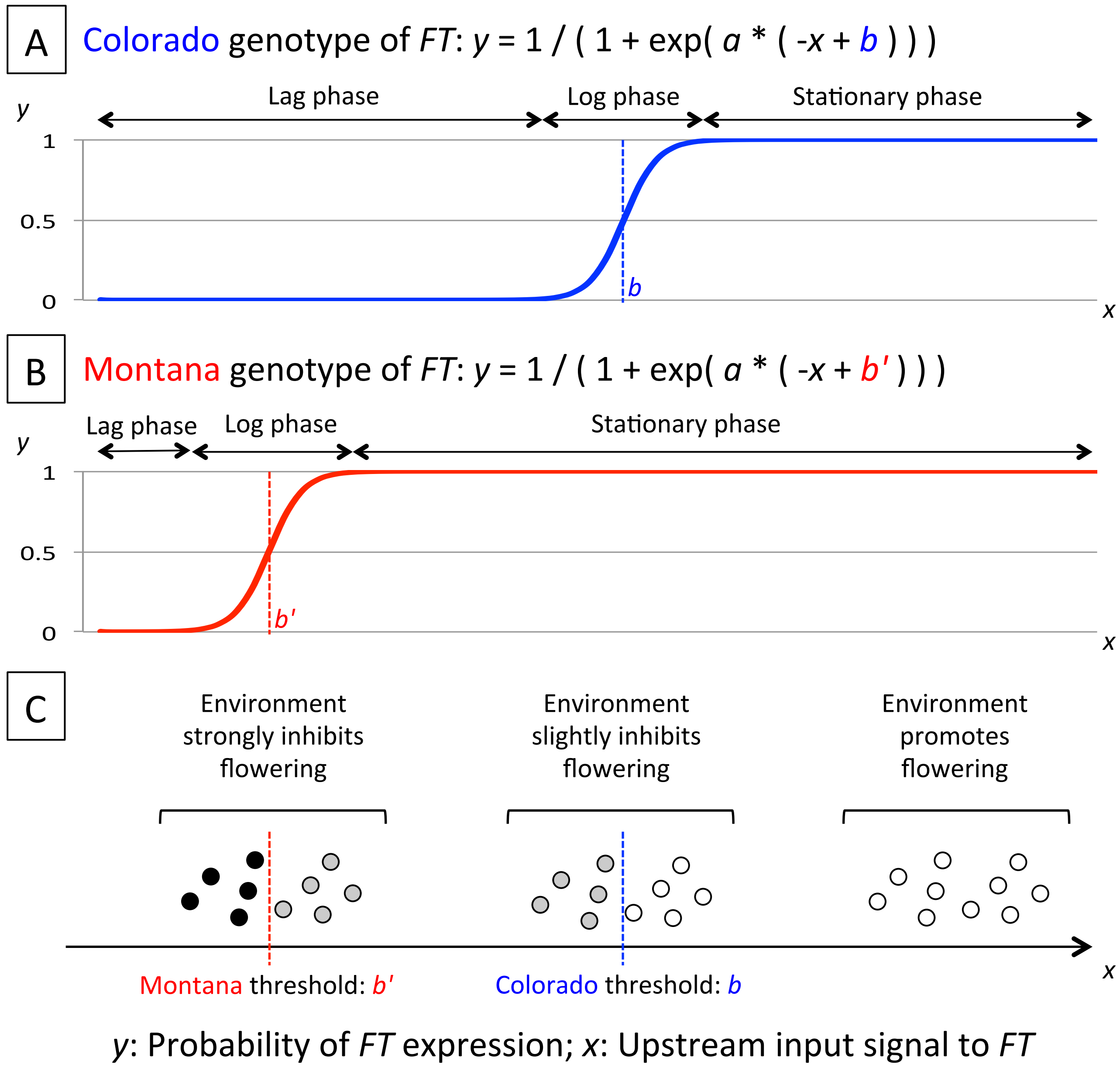

A
Colorado genotype of FT: y = 1 / (1 + exp(a * (-x + b)))
Lag phase
Log phase
Stationary phase
y
1
0.5
0
b
x
B
Montana genotype of FT: y = 1 / (1 + exp(a * (-x + b')))
Lag phase
Log phase
Stationary phase
y
1
0.5
0
b'
x
C
Environment strongly inhibits flowering
Environment slightly inhibits flowering
Environment promotes flowering
x
Montana threshold: b'
Colorado threshold: b
y: Probability of FT expression; x: Upstream input signal to FT
