# Supplementary material for: Unifying Genetic Canalization, Genetic Constraint, and Genotype-by-Environment Interaction: QTL by Genomic Background by Environment Interaction of Flowering Time in Boechera stricta
Source: PLoS Genet. 2014 Oct 23;10(10):e1004727. doi: 10.1371/journal.pgen.1004727 (PMC4207664; doi:10.1371/journal.pgen.1004727)
Supplement: Table S1 — Pairwise genetic correlations (upper diagonal) and the P values (lower diagonal) between 12 phenological traits in this study. (DOCX) [file pgen.1004727.s010.docx]

Table S1. Pairwise genetic correlations (upper diagonal) ^a^ and the *P* values (lower diagonal) between 12 phenological traits in this study ^b^.

| FT.12H.  18C.4W. | 0.759 | 0.684 | 0.531 | 0.367 | 0.420 | 0.840 | 0.694 | 0.531 | 0.430 | 0.089 | 0.274 |
| --- | --- | --- | --- | --- | --- | --- | --- | --- | --- | --- | --- |
| < 0.001 | FT.12H.  18C.6W. | 0.600 | 0.589 | 0.291 | 0.451 | 0.626 | 0.905 | 0.429 | 0.481 | 0.015 | 0.219 |
| < 0.001 | < 0.001 | FT.16H.  18C.4W. | 0.419 | 0.376 | 0.370 | 0.602 | 0.581 | 0.825 | 0.318 | 0.151 | 0.293 |
| < 0.001 | < 0.001 | < 0.001 | FT.16H.  18C.6W. | 0.241 | 0.383 | 0.473 | 0.515 | 0.256 | 0.825 | 0.133 | 0.285 |
| < 0.001 | < 0.001 | < 0.001 | 0.001 | FT.16H.  25C.4W. | 0.472 | 0.331 | 0.265 | 0.300 | 0.218 | 0.521 | 0.400 |
| < 0.001 | < 0.001 | < 0.001 | < 0.001 | < 0.001 | FT.16H.  25C.6W. | 0.428 | 0.422 | 0.314 | 0.362 | 0.225 | 0.747 |
| < 0.001 | < 0.001 | < 0.001 | < 0.001 | < 0.001 | < 0.001 | LN.12H.  18C.4W. | 0.658 | 0.555 | 0.491 | 0.182 | 0.345 |
| < 0.001 | < 0.001 | < 0.001 | < 0.001 | < 0.001 | < 0.001 | < 0.001 | LN.12H.  18C.6W. | 0.542 | 0.496 | 0.114 | 0.281 |
| < 0.001 | < 0.001 | < 0.001 | < 0.001 | < 0.001 | < 0.001 | < 0.001 | < 0.001 | LN.16H.  18C.4W. | 0.286 | 0.283 | 0.349 |
| < 0.001 | < 0.001 | < 0.001 | < 0.001 | 0.004 | < 0.001 | < 0.001 | < 0.001 | < 0.001 | LN.16H.  18C.6W. | 0.210 | 0.337 |
| 0.242 | 0.840 | 0.047 | 0.081 | < 0.001 | 0.003 | 0.017 | 0.135 | < 0.001 | 0.006 | LN.16H.  25C.4W. | 0.493 |
| < 0.001 | 0.004 | < 0.001 | < 0.001 | < 0.001 | < 0.001 | < 0.001 | < 0.001 | < 0.001 | < 0.001 | < 0.001 | LN.16H.  25C.6W. |

a. Calculated as the Pearson’s correlation coefficient (*r*) from family mean trait values.

b. The twelve traits are the flowering time (FT) and leaf number when flowering (LN) in six environments, shown in diagonal. Each trait is denoted by four fields separated by periods (Trait. Day length. Ambient temperature. Vernalization length.). For example, FT.12H.18C.4W. represents flowering time in the chamber with 12 hour days, 18 degree C, and 4 weeks of vernalization.
