# Supplementary material for: Unifying Genetic Canalization, Genetic Constraint, and Genotype-by-Environment Interaction: QTL by Genomic Background by Environment Interaction of Flowering Time in Boechera stricta
Source: PLoS Genet. 2014 Oct 23;10(10):e1004727. doi: 10.1371/journal.pgen.1004727 (PMC4207664; doi:10.1371/journal.pgen.1004727)
Supplement: Table S2 — Trait loadings on the first two principal components for all traits, flowering time traits, and leaf number traits shown in Figure 4. (DOCX) [file pgen.1004727.s011.docx]

Table S2. Trait loadings on the first two principal components for all traits, flowering time traits, and leaf number traits shown in Figure 4

|  | All traits | | Flowering time | | Leaf number | |
| --- | --- | --- | --- | --- | --- | --- |
| Traits and environments | PC1 | PC2 | PC1 | PC2 | PC1 | PC2 |
| Flowering time, 16 hour days, 18**°**C, 4 week vernalization | -0.314 | -0.083 | -0.421 | -0.111 | - | - |
| Flowering time, 16 hour days, 18**°**C, 6 week vernalization | -0.285 | -0.123 | -0.382 | -0.304 | - | - |
| Flowering time, 12 hour days, 18**°**C, 4 week vernalization | -0.347 | -0.197 | -0.470 | -0.223 | - | - |
| Flowering time, 12 hour days, 18**°**C, 6 week vernalization | -0.332 | -0.283 | -0.456 | -0.307 | - | - |
| Flowering time, 16 hour days, 25**°**C, 4 week vernalization | -0.222 | 0.413 | -0.323 | 0.749 | - | - |
| Flowering time, 16 hour days, 25**°**C, 6 week vernalization | -0.281 | 0.306 | -0.379 | 0.436 | - | - |
| Leaf number, 16 hour days, 18**°**C, 4 week vernalization | -0.277 | 0.010 | - | - | 0.414 | -0.044 |
| Leaf number, 16 hour days, 18**°**C, 6 week vernalization | -0.281 | -0.080 | - | - | 0.413 | -0.141 |
| Leaf number, 12 hour days, 18**°**C, 4 week vernalization | -0.341 | -0.137 | - | - | 0.482 | -0.289 |
| Leaf number, 12 hour days, 18**°**C, 6 week vernalization | -0.335 | -0.238 | - | - | 0.455 | -0.372 |
| Leaf number, 16 hour days, 25**°**C, 4 week vernalization | -0.143 | 0.530 | - | - | 0.274 | 0.707 |
| Leaf number, 16 hour days, 25**°**C, 6 week vernalization | -0.240 | 0.482 | - | - | 0.378 | 0.505 |
