# Supplementary material for: Unifying Genetic Canalization, Genetic Constraint, and Genotype-by-Environment Interaction: QTL by Genomic Background by Environment Interaction of Flowering Time in Boechera stricta
Source: PLoS Genet. 2014 Oct 23;10(10):e1004727. doi: 10.1371/journal.pgen.1004727 (PMC4207664; doi:10.1371/journal.pgen.1004727)
Supplement: Table S3 — Statistical tests of the interaction effect (epistasis) between nFT and other flowering-time QTL identified in a previous study. (DOCX) [file pgen.1004727.s012.docx]

Table S3. Statistical tests of the interaction effect (epistasis) between *nFT* and other flowering-time QTL identified in a previous study

| Growth chamber | The other QTL ^a^ | nFT effect | The other QTL effect | Interaction effect |
| --- | --- | --- | --- | --- |
| 12 hour days, 18**°**C, 4 week vernalization | Bst029595 | *F* = 40.43; *P* < 0.001 | *F* = 9.92; *P* = 0.002 | *F* = 0.03; *P* = 0.854 |
| 12 hour days, 18**°**C, 6 week vernalization | Fdh_Song | *F* = 35.73; *P* < 0.001 | *F* = 8.13; *P* = 0.005 | *F* = 1.69; *P* = 0.195 |
| 12 hour days, 18**°**C, 6 week vernalization | Bst007412 | *F* = 46.31; *P* < 0.001 | *F* = 11.67; *P* < 0.001 | *F* < 0.01; *P* = 0.977 |
| 16 hour days, 18**°**C, 4 week vernalization | At2g36390 | *F* = 35.27; *P* < 0.001 | *F* = 10.70; *P* = 0.001 | *F* = 0.49; *P* = 0.483 |
| 16 hour days, 18**°**C, 4 week vernalization | Con6547 | *F* = 40.54; *P* < 0.001 | *F* = 6.53; *P* = 0.012 | *F* = 0.05; *P* = 0.828 |
| 16 hour days, 18**°**C, 4 week vernalization | Rd22F | *F* = 32.44; *P* < 0.001 | *F* = 9.14; *P* = 0.003 | *F* = 0.30; *P* = 0.584 |
| 16 hour days, 18**°**C, 4 week vernalization | At5g12970 | *F* = 29.65; *P* < 0.001 | *F* = 4.33; *P* = 0.039 | *F* = 0.55; *P* = 0.460 |
| 16 hour days, 18**°**C, 6 week vernalization | Bst007412 | *F* = 12.00; *P* < 0.001 | *F* = 0.68; *P* = 0.409 | *F* = 0.89; *P* = 0.347 |
| 16 hour days, 25**°**C, 6 week vernalization | Bst002609 | *F* = 36.29; *P* < 0.001 | *F* = 12.82; *P* < 0.001 | *F* = 0.33; *P* = 0.568 |
| 16 hour days, 25**°**C, 6 week vernalization | At2g36390 | *F* = 45.20; *P* < 0.001 | *F* = 11.40; *P* < 0.001 | *F* = 1.96; *P* = 0.164 |

a. These QTL with significant main effect on flowering time in each growth chamber were listed in Appendix 11 of ‘Anderson JT, Lee C-R, Mitchell-Olds T (2011) Life history QTLs and natural selection on flowering time in Boechera stricta, a perennial relative of Arabidopsis. Evolution 65: 771-787’
