# Supplementary material for: Unifying Genetic Canalization, Genetic Constraint, and Genotype-by-Environment Interaction: QTL by Genomic Background by Environment Interaction of Flowering Time in Boechera stricta
Source: PLoS Genet. 2014 Oct 23;10(10):e1004727. doi: 10.1371/journal.pgen.1004727 (PMC4207664; doi:10.1371/journal.pgen.1004727)
Supplement: Table S4 — Primer sequences used in this study. (DOCX) [file pgen.1004727.s013.docx]

Table S4. Primer sequences used in this study ^a^

| Name | Sequence | Gene | Location | Use |
| --- | --- | --- | --- | --- |
| Bs-FT-E | ACAACTAACACAGAAACCACCT | *FT* | 5’ UTR | Cloning FT coding sequence, forward |
| Bs-FT-F | AATCGATTATAAAGGAAGAAGCC | *FT* | 3’ UTR | Cloning FT coding sequence, reverse |
| Bs-FT-C | CCAGATGTTCCAAGTCCTAGC | *FT* | 2^nd^ exon | *FT* qPCR, forward |
| BsFT-CLqPCR-B | GCAGCCACTCTCCCTCTGAC | *FT* | 4^th^ exon | *FT* qPCR, reverse |
| BsACT2-CL-C | CGTACAACCGGTATTGTGCTG | *ACT2* | 1^st^ and 2^nd^ exon, spanning intron | *ACT2* qPCR, forward |
| BsACT2-CL-A | TGCTGTTGTGGTGAACATGTAA | *ACT2* | 2^nd^ exon | *ACT2* qPCR, reverse |

a. The *FT* gene (AT1G65480) has a sister copy, *TWIN SISTER OF FT* (*TSF*, AT4G20370). The *FT* primers are designed to be conserved between the *FT* orthologs in *Arabidopsis* and *Boechera* but divergent from *TSF*.
