# Supplementary material for: Unifying Genetic Canalization, Genetic Constraint, and Genotype-by-Environment Interaction: QTL by Genomic Background by Environment Interaction of Flowering Time in Boechera stricta
Source: PLoS Genet. 2014 Oct 23;10(10):e1004727. doi: 10.1371/journal.pgen.1004727 (PMC4207664; doi:10.1371/journal.pgen.1004727)
Supplement: Figure S4 — QTL controlling the covariance structure of phenological traits in growth chamber environments. Each row represents a statistical method on a group of traits across several environments. For example, ‘Box's M, all traits’ represents the results of Box's M method on flowering time and leaf number from all six environments (12 traits total), and ‘Krzanowski, leaf number’ represents results of Krzanowski method on leaf number in six environments. Each column represents a genetic marker on the linkage map, and chromosomes are separated by vertical white lines. Black cells represent markers significantly controlling the covariance of a group of traits, whereas dark grey cells are non-significant markers. Light grey cells are markers that are excluded due to segregation distortion (see main text). (PDF) [file pgen.1004727.s004.pdf]

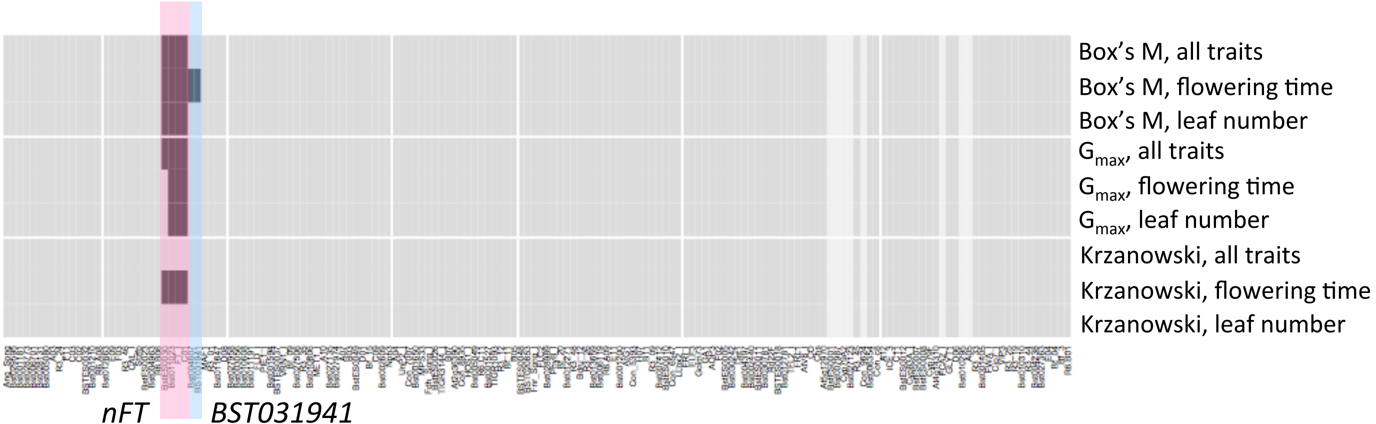

Box's M, all traits
Box's M, flowering time
Box's M, leaf number
G_{max}, all traits
G_{max}, flowering time
G_{max}, leaf number
Krzanowski, all traits
Krzanowski, flowering time
Krzanowski, leaf number
nFT
BST031941
